# A Review on Cementitious and Geopolymer Composites with Lithium Slag Incorporation

**DOI:** 10.3390/ma17010142

**Published:** 2023-12-27

**Authors:** Hongxiang Gou, Madhuwanthi Rupasinghe, Massoud Sofi, Rajesh Sharma, Gianluca Ranzi, Priyan Mendis, Zipeng Zhang

**Affiliations:** 1 Department of Infrastructure Engineering, The University of Melbourne, Parkville 3053, Australia; hongxiangg@student.unimelb.edu.au (H.G.); madhuwanthi.rupasinghearach@unimelb.edu.au (M.R.); massoud@unimelb.edu.au (M.S.); pamendis@unimelb.edu.au (P.M.); 2 Tianqi Lithium Energy Australia, Kwinana 6167, Australia; rajesh.sharma@tianqilithium.com.au; 3 Centre for Advanced Structural Engineering, The University of Sydney, Sydney 2006, Australia; gianluca.ranzi@sydney.edu.au

**Keywords:** lithium slag, cement, concrete, composite, durability, strength, hydration, carbon footprint, review

## Abstract

This study critically reviews lithium slag (LS) as a supplementary cementitious material (SCM), thereby examining its physiochemical characteristics, mechanical properties, and durability within cementitious and geopolymer composites. The review reveals that LS’s particle size distribution is comparable to fly ash (FA) and ground granulated blast furnace slag (GGBS), which suggests it can enhance densification and nucleation in concrete. The mechanical treatment of LS promotes early hydration by increasing the solubility of aluminum, lithium, and silicon. LS’s compositional similarity to FA endows it with low-calcium, high-reactivity properties that are suitable for cementitious and geopolymeric applications. Increasing the LS content reduces setting times and flowability while initially enhancing mechanical properties, albeit with diminishing returns beyond a 30% threshold. LS significantly improves chloride ion resistance and impacts drying shrinkage variably. This study categorizes LS’s role in concrete as a filler, pozzolan, and nucleation agent, thereby contributing to the material’s overall reduced porosity and increased durability. Economically, LS’s cost is substantially lower than FA’s; meanwhile, its environmental footprint is comparable to GGBS, thereby making it a sustainable and cost-effective alternative. Notwithstanding, there is a necessity for further research on LS’s fine-tuning through grinding, its tensile properties, its performance under environmental duress, and its pozzolanic reactivity to maximize its utility in concrete technologies. This study comprehensively discusses the current strengths and weaknesses of LS in the field of building materials, thereby offering fresh perspectives and methodologies to enhance its performance, improve its application efficiency, and broaden its scope. These efforts are driving the sustainable and green development of LS in waste utilization and advanced concrete technology.

## 1. Introduction

Lithium, known for its exceptional electrochemical properties, is extensively used across various industries, including pharmaceuticals [1], lubrication [2], glass [3], and ceramics [4]. The late 1990s marked a significant surge in lithium demand due to its crucial role in portable electronic battery production [5]. These batteries have become indispensable for modern life, thereby providing clean energy for smart devices and vehicles [6]. Spodumene, the primary raw material for lithium batteries, is mainly found in limited deposits globally, with Australia ranking second in lithium reserves after Chile, thereby notably dominating the global battery market.

During the production of lithium carbonate from spodumene, chemical treatment yields lithium slag (LS) and metal oxides [7,8]. Approximately 9–10 tons of LS are generated per ton of lithium carbonate produced, thus posing disposal challenges [9,10]. In 2022, Australia contributed 68,500 tons to global lithium production, and demand has been projected to increase from 114,400 tons in 2022 to 248,400 tons in 2026 [11]. Improper disposal of LS could harm the environment due to the leaching of fluorine (F^−^) and sulphate (SO_4_^2−^) ions, thereby requiring careful management [12].

LS, which is rich in silicon, aluminum, and calcium oxides, has garnered interest as a substitute for ordinary Portland cement (OPC) in concrete production, thereby offering a reduced carbon footprint and costs [13,14]. However, its sulfuric acid extraction origins with elevated SO_3_ levels necessitate caution in its use to maintain cement stability [15,16,17,18]. Adopting a prudent approach is vital to leverage LS sustainability without compromising the cement matrix.

Considerable research efforts have been dedicated to the utilization of LS in cementitious composites, particularly in China and Australia. Recent investigations conducted by [19,20,21] have collectively affirmed LS’s potential as a supplementary cementitious material and as a partial precursor for the formulation of alkali-activated geopolymers. This is underpinned by the pozzolanic activity exhibited by LS, as well as its capacity to enhance the pore structure of cementitious materials. As shown in Table 1, numerous studies have incorporated LS into binary, ternary, and even quaternary systems in conjunction with other cementitious materials to fabricate mortars, concrete, and geopolymers. Furthermore, the application of LS has been explored in alkali-activated geopolymers [5,16,22,23,24], backfill materials [25,26], ceramsite production [27], and brick manufacturing [28]. These diverse applications underscore the versatility and potential of LS as a valuable resource in various construction and material science endeavors.

To gain insight into the potential applications of LS and to enhance its utilization efficiency, it is imperative to comprehensively understand the physicochemical properties inherent to LS itself. Presently, numerous studies have embarked on an exploration of the physicochemical properties of LS. Research has demonstrated that particle size, chemical composition, and amorphous content (silica and alumina) are key determinants of the pozzolanic activity exhibited by noncrystalline materials [40]. Therefore, investigations into the physical characteristics of LS encompass parameters such as the specific surface area (SSA) [14,41], specific gravity [33], density [42,43], moisture content [41], and particle size distribution [22,44]. In parallel, conventional analytical methods have been employed to scrutinize the chemical composition of LS, including X-ray diffraction (XRD) [23,44] and scanning electron microscopy with energy-dispersive spectroscopy (SEM/SEM-EDS) [44,45,46], as well as specialized techniques such as Fourier transform infrared spectroscopy (FTIR) [31,45], nuclear magnetic resonance (NMR) [30,47], X-ray photoelectron spectroscopy (XPS) [30], and thermogravimetry (TG)/thermogravimetric and derivative thermogravimetric analysis (TG-DTG) [19,43,48].

Due to its analogous physicochemical properties to SCMs, LS has been widely adopted as a cementitious material additive in mortar and concrete. Extensive research has been conducted on the performance of cementitious composite materials incorporating LS, including aspects such as setting times [29,34], flowability [49], and rheology [16,50]. Furthermore, the mechanical properties of cementitious materials with LS incorporation have been thoroughly investigated, thus encompassing parameters like compressive strength [50,51], tensile strength [52,53], elastic modulus [54], and flexural strength [49]. Moreover, the durability of LS-based cementitious composites has received significant attention, including studies on chloride migration [55,56], sulfate attack [57,58], shrinkage [56,59], freeze–thaw resistance [57], acid corrosion [60], and creep [50]. Furthermore, the utilization of LS in the construction materials industry not only mitigates environmental pollution but also significantly enhances economic benefits. Therefore, the economic and environmental benefits of incorporating LS into cementitious composites have been assessed, thereby encompassing cost analysis, carbon emissions, and energy consumption [5,61].

This review provides a comprehensive analysis of the physiochemical, mechanical, durability, and microstructural properties of LS-based cementitious composites. Additionally, it incorporates an in-depth examination of the economic and environmental implications associated with LS utilisation in cementitious composites. Based on the findings, this research offers valuable recommendations for the sustainable incorporation of LS in concrete, and it identifies key areas requiring future research endeavors.

## 2. Physiochemical and Microscopic Analysis of LS

In this section, a review of the physiochemical and microstructural properties of LS is conducted. Understanding LS’s physical, chemical, and microscopic properties is paramount to harness its potential applications. Firstly, the physical properties of LS, thereby encompassing aspects such as particle size distribution (PSD), density, specific surface area (SSA), and moisture content, have been scrutinized. In terms of the chemical properties, XRD and X-ray fluorescence (XRF) have been utilized to uncover its elemental composition and structure. Microscopic analysis, facilitated by SEM, TG, XPS, and NMR spectroscopy, has offered insights into the molecular-level characteristics of LS. This groundwork research is essential for a comprehensive exploration of LS’s potential applications across various domains.

### 2.1. Physical Properties of Raw LS

#### 2.1.1. Particle Size Distribution

PSD is a crucial physical parameter used to assess the activity index of LS. PSD is typically characterized by its 10% (D10), 50% (D50), and 90% (D90) percentiles, representing the respective sizes finer than the nominated percentile. Variations in LS particle sizes are evident across different studies, as are shown in Table 2. For instance, the D10 of LS has been observed to range from 0.84 to 2.74 µm, with an average value of 2.01 µm. Similarly, the D50 (mean particle size) exhibited a range of 4.53–30.39 µm and an average value of 17.04 µm. Furthermore, the D90 was reported in the range of 28.00–83.65 µm, with an average size of 58.86 µm. These variations primarily depend on the source of the LS, roasting temperature, and the grinding methods and procedures [62]. Unfortunately, there is limited literature on systematic studies of the grinding methods and grinding parameters that can enhance the reactivity of LS. In response to the inherent characteristics of raw LS particles, which are characterized by their large size and low amorphous content, limited research endeavors have undertaken the examination of the grinding processes, both dry [14] and wet [29,30], to reduce particle size and enhance amorphousness. Wet grinding, in particular, has been effective in producing finely ground LS particles that facilitate ion dissolution. The incorporation of wet-ground LS has shown promise in improving the early strength of sulfoaluminate cement (SAC) [29]. Moreover, wet grinding for 30 min has demonstrated remarkable grinding efficiency, thus reducing the D(50) of LS from 30.38 µm to 3.04 µm. This process significantly enhanced the dissolution of aluminum, lithium, and silicon in LS, thereby contributing to the expedited early hydration of the SAC-LS system [29]. This improvement primarily stems from the nucleation-inducing effect on hydration, the refinement of pore structure, and the heightened pozzolanic reactivity of micro-LS [30].

**Figure 1:**
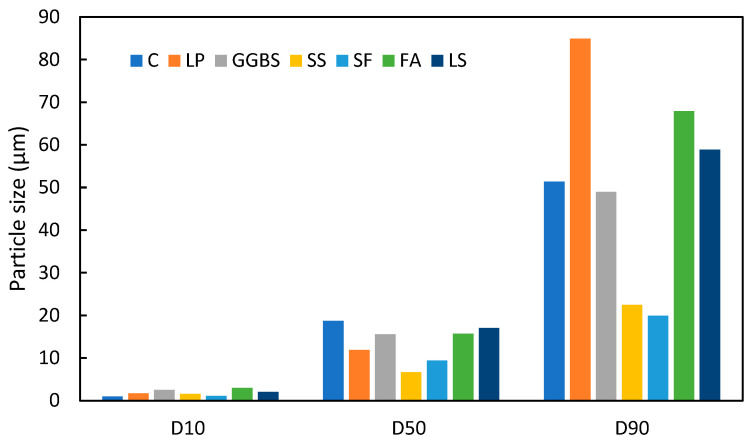
Particle sizes of different cementitious materials (C [62], LP [66], GGBS [46], SS [67], SF [68], FA [69]).

To investigate the differences in particle size between LS and other commonly used SCMs, the average values of the D10, D50, and D90 from Table 2 were compared with those of conventional cement and SCMs. The particle size distributions of various cementitious materials are depicted in Figure 1 based on different literature sources. It is evident that the PSD of LS is comparable to that of FA and GGBS. This size similarity suggests that LS can exhibit similar densification and nucleation effects when incorporated into concrete that are akin to FA and other SCMs [64,65].

#### 2.1.2. Density, Specific Surface Area, and Moisture Content

In addition to analyzing the PSD of LS, various physical properties, including density, SSA, and moisture content, have also been examined. The SSA of LS, as reported by different researchers [14,38,42], exhibited a range from 265 to 1362 m^2^/kg, with an average value of 698 m^2^/kg, which is attributed to the porous surface texture of LS [70]. It is generally finer than the SSA of cement (350 m^2^/kg), fly ash (350 m^2^/kg), and GGBFS (400 m^2^/kg) [71,72,73]. The density of LS was found to fall within the range of 2480–2500 kg/m^3^ [43,57]. The moisture content in LS varied from 2.5% to 28.3%, with an average value of 17.5% based on different studies [45,70]. These physical properties of LS are closely linked to the preparation methods and grinding parameters of LS. To achieve good quality control and high use efficiency of LS, further research into the manufacturing methods and production parameters of LS is essential.

### 2.2. Chemical Properties of LS

#### 2.2.1. Chemical Composition

The chemical composition analysis of LS serves to provide fundamental data for assessing its potential as a cementitious material. Numerous studies have investigated the chemical composition of LS through XRF testing, as are depicted in Table 3. The analysis reveals that the primary chemical components of LS are SiO_2_, Al_2_O_3_, and CaO. SiO_2_ and Al_2_O_3_ are predominantly present in the form of H_2_O·Al_2_O_3_·4SiO_2_ (aluminum silicate), while CaO primarily exists as CaSO_4_ (anhydrous gypsum) [74]. Furthermore, LS exhibits a notable sulfur trioxide (SO_3_) content, thereby typically existing in the form of CaSO_4_·2H_2_O, which, upon reaction with calcium hydroxide, can lead to expansion. This limitation prevents the substantial inclusion of LS as a mineral admixture [75]. Interestingly, in numerous pieces of literature, the chemical composition does not mention the content of Li_2_O. This is primarily because the Li_2_O content in LS is generally less than 1%, and Li_2_O is not considered to be a significant influencing factor on the performance of LS [39].

**Figure 2:**
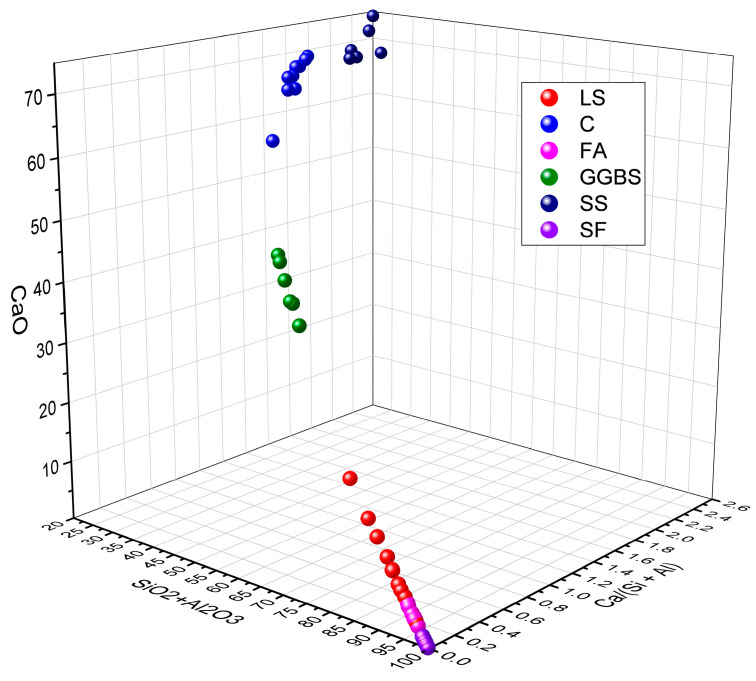
The ranges of SiO_2_ + Al_2_O_3_, CaO, and Ca/(Si + Al) in various cementitious materials.

Moreover, the ratios of SiO_2_ + Al_2_O_3_ and CaO, as well as the Ca/(Si + Al) value, reflect its potential as an SCM and reveal differences in the pozzolanic reactivity and hydration activity. By analyzing the data presented in Table 3, the ranges of the SiO_2_ + Al_2_O_3_, CaO, and Ca/(Si + Al) of LS in comparison with C, FA, GGBS, SS, and SF were determined, as are shown in Figure 2 [76,77,78,79]. From this comprehensive analysis, it is evident that the composition of SiO_2_ + Al_2_O_3_, CaO, and Ca/(Si + Al) in the relevant literature exhibits considerable variations, thereby falling within the specified ranges of 17.7–97.6%, 2.2–62.9%, and 0.02–1.94%, respectively. These disparities manifest significant distinctions in the performance characteristics of cementitious materials. Specifically, when focusing on LS, the parameters SiO_2_ + Al_2_O_3_, CaO, and Ca/(Si + Al) fall within the narrower ranges of 70.3–80.7%, 2.5–18.5%, and 0.04–0.14%, respectively. It is apparent that, in comparison to C, where the Ca/(Si + Al) value typically resides around 1.4, a Ca/(Si + Al) value significantly below one in LS signifies the potential presence of pozzolanic reactivity in the LS, rather than predominantly indicating hydration reactivity [80]. In addition, upon comparing the chemical compositions of LS with those of commonly used cementitious materials, as are demonstrated in Figure 2, it becomes evident that LS shares compositional similarities with FA, which are characterized by high SiO_2_ and Al_2_O_3_ contents while exhibiting a low CaO content and Ca/(Si + Al) values. This resemblance categorizes LS as a low-calcium precursor with pozzolanic reactivity akin to that of FA [81].

#### 2.2.2. XRD Results

**Figure 3:**
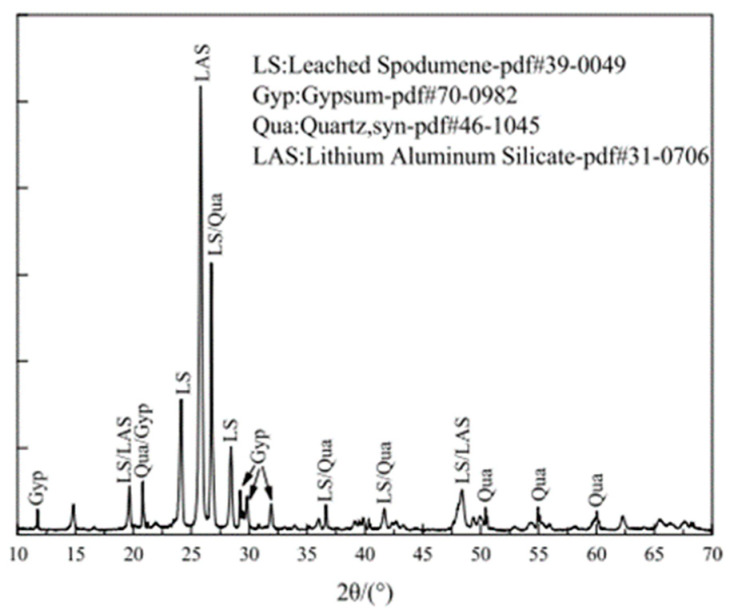
XRD patterns of LS powder [45].

XRD analysis provides a quantitative assessment of the mineral composition of LS. According to references [34,39,45], the primary mineral phases in LS have been identified through XRD patterns, as are illustrated in Figure 3. These phases mainly include gypsum, quartz, and lithium aluminum silicate.

Quantitative analysis conducted by Rahman et al. [19] revealed the relative compositions of the crystalline mineral phases in LS. The analysis indicated that LS comprises 2.1% β-spodumene (LiAlSi_2_O_6_), 6.6% bassanite (CaSO_4_·0.5H_2_O), 5.4% calcium magnesium carbonate (Ca_0.845_Mg_0_._155_(CO_3_)), 23.8% quartz (SiO_2_), 28.2% anorthite (CaAl_2_Si_2_O_8_), 2.3% albite (NaAlSi_3_O_8_), and 31.6% amorphous phase. Furthermore, the research conducted by Zhai et al. [45] demonstrated that LS predominantly contains aluminosilicates in the form of spodumene, anorthite, and albite. Traces of aluminosilicates were also found in orthoclase (KAlSi_3_O_8_), while silica was predominantly present as quartz (SiO_2_). Additionally, the calcium content was primarily associated with calcite (CaCO_3_), with traces detected in anhydrite, actinolite (Ca_2_Si_8_O_22_(OH)_2_), and dolomite (CaMg(CO_3_)_2_). Karrech et al. [82] observed the presence of several minerals including quartz, bassanite/gypsum, and orthoclase (K-feldspar). The rest was leached pyroxene that is believed to contain amesite and pyrophyllite. The particularity of those minerals is that they contain chemically bound water and hydroxyl groups in their structures, which can be lost upon igniting the material to 1000 °C.

### 2.3. Microscopic Analysis of LS

#### 2.3.1. SEM-EDS Analysis

**Figure 4:**
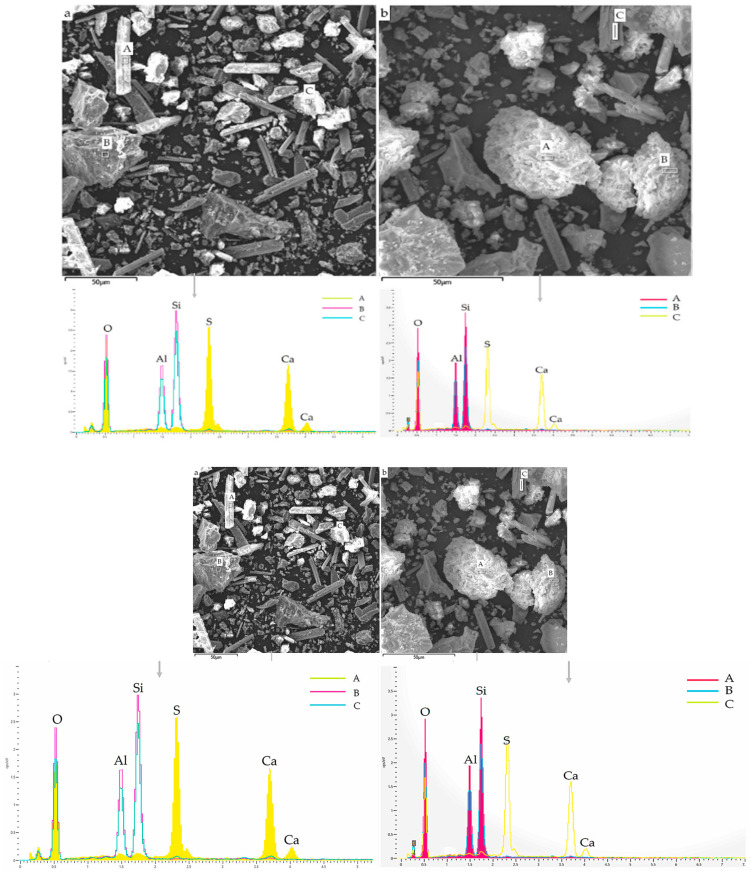
SEM-EDS results: (**a**) raw LS and (**b**) calcined LS [23].

Javed et al. [23] employed SEM-EDS to explore the phase and morphological change between raw LS and calcined LS. LS comprises angular particles that are rich in aluminosilicate minerals, while the prismatic/elongated particles consist of gypsum, as is evidenced by the SEM/EDS analysis depicted in Figure 4a. The EDS points B and C on the micrograph exhibited peaks corresponding to aluminum, silicon, and oxygen EDS spectra, thereby confirming the presence of aluminosilicate particles. Conversely, EDS analysis of the prismatic particles shown in Figure 4b displayed peaks of calcium, sulfur, and oxygen, thus indicating the presence of gypsum. This observation supports the notion of sulfation occurring during lithium extraction in refineries [83]. These prismatic particles possess a size exceeding 50 μm. Through the calcination of LS, the aluminosilicate components undergo a transformation into agglomerated amorphous (glassy) phases due to sintering. Particle fragmentation is a consequence of the lithium refining process being applied to spodumene ore, which induces a certain level of reactivity within LS. Notably, the crystalline phase transformation within LS initiates from temperatures exceeding 800 °C [84]. Thus, a calcination temperature of 700 °C is considered to be suitable for the production of amorphous aluminosilicates [23]. Moreover, an approach combining thermal treatment with chemical activation was employed in the study of Li et al. [31] to enhance the reactivity of LS. The outcomes demonstrated that preheating effectively promoted the reactivity of the LS, thereby leading to a significant increase in the proportion of active amorphous components in the LS, which surged from 17.3 wt% to 50.7 wt% following heat activation at 700 °C. Karrech et al. [82] observed DβS, gypsum needles measuring about 20 μm in length, larger aluminosilicate particles, and other minerals, thereby suggesting an aluminosilicate grain with parallel cracks in it; such parallel cracks form during β-spodumene leaching due to Li-H exchanges.

#### 2.3.2. TG-DTG Analysis

**Figure 5:**
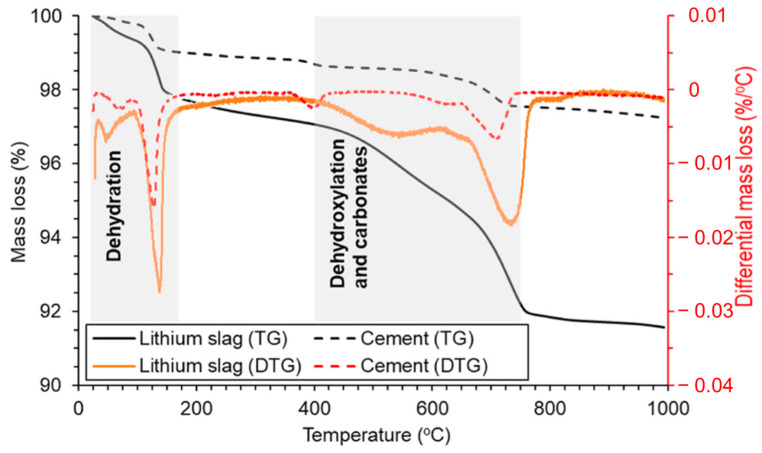
TG-DTG curves of LS and cement from room temperature to 990 °C [19]. Note: the shaded areas in the TG-DTG curves show the mass loss due to presence of moisture and gypsum, portlandite, and carbonates, respectively.

TG-DTG testing provides valuable insights into the thermal behavior of LS. It allows researchers to understand how LS reacts to changes in temperature, thus helping to identify important thermal transitions and decomposition processes. TG-DTG curves for LS compared with cement were obtained by Rahman et al. [19], as are depicted in Figure 5.

The observed mass loss in the TG-DTG curves of the LS, occurring between room temperature and 170 °C, primarily resulted from the removal of moisture and bassanite water [85]. It is evident that the moisture content of LS was notably higher in comparison to conventional cement. Subsequent mass losses in the temperature range of 400–750 °C can be attributed to the dehydroxylation of the zeolite phases [86] and the decomposition of carbonates [65]. These processes contribute to the reduction in the crystallinity of pozzolans. During this stage, there is a substantial decrease in the mass of LS, thus indicating a continuous augmentation of the amorphous phases within LS, which can significantly enhance its pozzolanic reactivity [87]. This research corroborates the previous conclusion that the most optimal temperature for enhancing the reactivity of LS is approximately around 700 °C [23,31]. It is noteworthy that the higher moisture content in LS compared to cement, coupled with the more pronounced amorphous reactions, resulted in a total mass loss of the LS reaching 8.4% at 990 °C. Importantly, the LS retained the 31.6% proportion of amorphous phases confirmed by XRD analysis, which was primarily composed of aluminosilicate glassy phases. The comprehensive analysis of the combined results from XRD and TG-DTG tests suggests that these amorphous phases have the potential to participate in pozzolanic reactions during the hydration of cement particles. Consequently, it is valuable to assess the pozzolanic activity of LS through various pozzolanic activity tests.

#### 2.3.3. NMR and XPS Analysis

NMR patterns provide insights into the chemical environment surrounding silicon-oxygen tetrahedra, while XPS analysis yields binding energies that also reflect the chemical environment of the atoms. Therefore, Tan et al. [30] employed microscopic techniques such as XPS and NMR to investigate the chemical composition changes in LS as it transformed into micro-LS through wet grinding.

The NMR results revealed a noticeable change: the peak ranging from −110 ppm to −90 ppm, indicative of the Si-O tetrahedron, was prominent in LS but significantly diminished in micro-LS. This phenomenon was attributed to the mechanical forces applied during wet grinding or the self-hydration reactions of silicates, thereby altering the chemical environment of the Si-O tetrahedron. On the other hand, XPS results showed that wet grinding reduced the binding energy for silicon from 101.30 eV in the LS to 100.93 eV, as well as for aluminum and calcium, which shifted from 73.19 eV to 72.9 eV and from 346.81 eV to 346.48 eV, respectively. The decrease in binding energy suggests that during the wet grinding process, the silicates, aluminates, and calcium in LS could dissolve into a liquid phase, thus improving its pozzolanic activity.

## 3. Fresh State Properties of Cementitious Composites with LS Incorporation

This section reviews the reported literature on critical aspects of the fresh state properties of cementitious composites incorporating LS. This includes the setting time, flowability, and rheology. These factors are pivotal in determining the practical utility of LS in various applications. Setting time influences the temporal aspects of LS handling, flowability affects its ease of transport and placement, and rheology defines its flow behavior and viscosity. Understanding and optimizing these parameters are essential for harnessing the full potential of LS in the construction materials field, particularly as a potential supplementary cementitious material.

### 3.1. Setting Time

**Figure 6:**
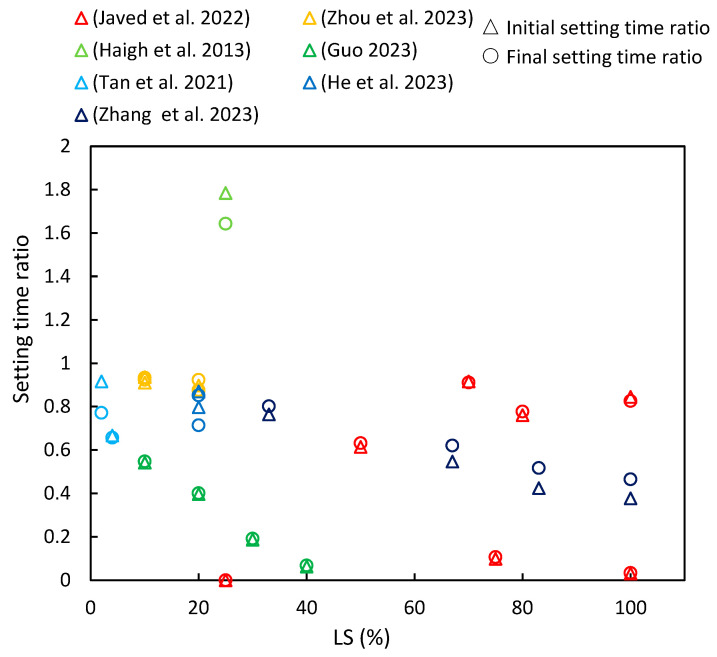
The setting time ratios of cementitious composites with varying levels of LS substitution [16,23,37,39,41,63,88].

This section compiles data from various references on the changes in initial and final setting times with increasing LS content, as are depicted in Figure 6. The setting time ratio is defined as the ratio of the setting time of the cementitious composite with added LS to the setting time of the corresponding sample without LS (control group), with the subsequent parameter ratios described later on in this paper (including flowability, strength characteristics, chloride resistance, and shrinkage) being defined in the same manner. It is evident that the majority of cementitious composites incorporating LS exhibited a setting time ratio of less than one, thereby indicating that the addition of LS reduces the setting time of cementitious composites. Furthermore, for most of the literature reviewed, an increase in LS content further decreased the setting time. Moreover, the similarity between the initial setting time ratio and the final setting time ratio indicates an analogous trend between the initial and final setting times.

This section provides a detailed analysis of these specific trends. Zhang et al. [37] observed that as the proportion of LS replacing LP increased, both the initial and final setting times of the paste decreased. In LS-UHPC (ultra-high-performance concrete), the increased formation of ettringite (AFt) with higher LS ratios contributed to reduced setting times by facilitating the solid network’s overlap reduction [89]. Javed et al. [23] investigated the setting times of geopolymer pastes containing LS and increasing amounts of FA. Their findings indicated an increase in both the initial and final setting times with higher FA replacement. The abrupt setting observed in the LS geopolymer was attributed to false setting due to the presence of over 5% gypsum/anhydrite in the LS. This phenomenon was linked to the precipitation of interlocked needle-shaped gypsum (anhydrite), which contributed to self-desiccation in the geopolymer paste matrix due to reduced water content in the aluminosilicate gel [90]. In addition, Javed et al. [23] studied the setting time of an LS-SF geopolymer paste at various Si/Al ratios. They observed that the initial setting time increased by over 20% at a Si/Al ratio of 3.5, thereby indicating the significant suppression of secondary anhydrite formation. In the LS-SF geopolymer, the setting accelerated at a Si/Al ratio of 3.5 and decelerated with higher Si/Al ratios, thereby demonstrating the influence of silica fume on the setting time. Moreover, Zhou et al. [63] concluded that as LS content increased from 0% to 20%, the initial and final setting times of fresh mortar decreased by 16.7% and 12.1%, respectively. A previous study also supported that LS addition can expedite the setting time by accelerating ettringite formation, which is a major hydration product of cement [16]. However, Haigh et al. [41] conducted experiments using concretes with a strength grade of 40 MPa and 25% LS as an SCM and found that the initial and final setting times were 580 and 690 min, respectively, which exceeded those of the control specimens.

Furthermore, various researchers have conducted in-depth investigations into the influence of LS on the setting time in special cementitious systems. Guo et al. [16] conducted a study in which ternary alkali-activated materials (AAMs) were synthesized using a combination of LS, metakaolin (MK), and GGBS. They explored the impact of LS replacing MK on the setting time. As the LS content increased, there was a significant reduction in the setting time. This phenomenon was attributed to the LS requiring less time for the dissolution of (Si, Al)O_4_ tetrahedra and Ca^2+^ ions in the same alkali activator, thereby creating more favorable conditions for polymerization and resulting in a shortened setting time. These findings were supported by Tan et al. [88] in the context of the SAC system, where the setting process relied on calcium sulfoaluminate hydration and AFt formation. Nano-LS prepared via wet grinding was found to markedly enhance the early hydration process and AFt formation. The presence of dissolved lithium salt in LS expedited the formation of the AFt due to SAC usage, thus often eliminating the induction period [91]. Additionally, He et al. [39] demonstrated the synergistic impact of C-S-H-PCE and TEA on the setting behavior of LS-blended cement. The combined use of C-S-H-PCE and TEA altered the setting of LS–cement binders, with a strong correlation observed between the dosage of C-S-H-PCE and TEA and the setting behavior.

### 3.2. Flowability

**Figure 7:**
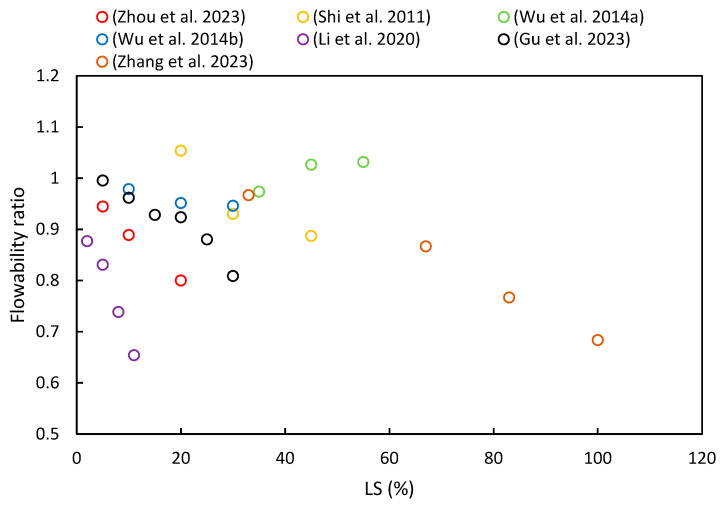
The flowability ratios of cementitious composites with varying proportions of LS [24,37,49,52,53,63,92].

The flowability of fresh paste is primarily characterized through slump tests and flow table tests. Figure 7 summarizes the flowability ratios of cementitious composites with varying proportions of LS based on different literature sources. It is evident from most of the literature that flowability decreases with an increase in LS content. Specific details of these changes are as follows.

Zhang et al. [37] noted that the addition of LS reduced the flowability of UHPC due to its irregular shape and strong water absorption. Additionally, LS led to increased AFt formation in the initial stages of hydration, which has been attributed to its high sulfate and aluminate content [45]. Zhou et al. [63] also observed that the flowability decreased as the LS content increased: 85 mm at 5%, 80 mm at 10%, and 72 mm at 20%. This decline in flowability has been attributed to LS’s high surface roughness, large specific surface area, and irregular particle shape [93]. Moreover, Luo et al. [24] investigated the effect of activators on the flowability of LS-based geopolymer binders. The NaOH + Na_2_SiO_3_ binder had the highest flowability at 192 mm, while the NaOH + CaCO_3_ binder had the lowest at 188 mm, though the differences were relatively minor due to the low activator content. Li et al. [49] found that the flowability of white reactive powder concrete decreased with increasing LS content, thereby ranging from 0% to 11% as an SCM. The flow values decreased from 260 to 170 mm, which was attributed to the high specific surface area of the primary cubicite crystalline phase in the LS, thereby increasing the water demand in the slurry. Furthermore, Wu et al. [92] utilized LS and FA as SCMs for high-performance concrete, where the slump decreased as the LS content increased. With an LS cement substitution rate ranging from 0% to 30%, the slump decreased from 186 mm to 175 mm, respectively. These findings align with previous research [53,82].

However, some studies have shown different patterns. Wu et al. [52] conducted research on concrete slumps using water-to-binder ratios ranging from 0.27 to 0.35 and a ternary combination of 25–65% OPC, 15–35% LS, and 20–40% SS content. The sand ratio and superplasticizer dosage were adjusted to maintain the concrete mixture’s slump within the range of 190 ± 20 mm. Due to the numerous variables involved, the slump values did not exhibit a certain trend with the LS contents. Furthermore, He et al. [36] studied concrete with LS contents ranging from 40% to 60% and reported a reduction in workability as the LS content increased. To achieve satisfactory flowability, this study increased the dosage of the superplasticizer, thus keeping the slump value within the range of 180 to 200 mm [94]. In addition, Shi et al. [53] reported that the slump values of high-performance LS concrete initially increased and then decreased with increasing LS substitution for cement. For a 20% LS substitution rate, the slump reached a maximum of 196 mm but then sharply decreased to 165 mm with a 45% LS dosage. Lastly, Gu et al. [95] observed that the addition of SS increased the flowability of blended mortar, while the addition of LS reduced the flowability. Overall, the flowability of fresh mortar gradually decreased with an increasing LS/SS ratio.

### 3.3. Rheology

Rheology, which is a branch of science focused on the deformation and flow of matter, explores the relationships between the stress, strain, and shear rate. In the context of cementitious materials, the rheological behavior of fresh mixtures plays a significant role in determining their optimal mixing, casting, and stacking properties. This is especially crucial in specialized construction techniques like 3D printing and self-compacting concrete [96,97,98].

He et al. [39] noted that an increased dosage of synthetic calcium silicate hydrates–polycarboxylate (CSH-PCE) led to a notable enhancement in the rheological properties of fresh LS–cement paste. This enhancement was evident in decreased viscosity and yield stress. It resulted from the physically combined PCE in C-S-H-PCE, which could dissolve in the solution and then adsorb onto minerals, thereby improving the flowability of the fresh paste. However, the addition of TEA had a negative impact on the rheological properties of the LS–cement paste. The incorporation of 1% C-S-H-PCE into the LS–cement binder, along with increasing the TEA content (from 0% to 0.5%), progressively increased the yield stress and viscosity values of the fresh binder. Furthermore, the influence of Na_2_SO_4_ on the rheological performance of LS binders was explored by He et al. [99]. The results indicated that Na_2_SO_4_ adversely affected the viscosity properties of LS–cement binders. An increased Na_2_SO_4_ dosage resulted in heightened viscosity properties and reduced flow behaviors of LS–cement binders. This effect was attributed to the accelerating impact of Na_2_SO_4_ on the hydration process, thereby leading to increased hydrate content and expedited development of the hardened microstructure. Additionally, the rheological characteristics were well fitted using the Herschel–Bulkley model, while the Bingham model accurately described all of the system [100]. Rahman et al. [70] employed various rheological models, including the Bingham, modified Bingham, and Herschel–Bulkley models, to characterize the yield stress and plastic viscosity values of LS cement pastes across different volume fractions. Their study suggested that a 40% LS cement paste is a viable option for producing green concrete with optimal fresh state and rheological properties. Tian et al. [101] investigated the impacts of low surface free energy mineral admixtures, such as CaF_2_-dominated fluorite and LS, on the rheological behavior of alkali-activated slag (AAS) pastes. As the LS content increased from 0 to 30 wt%, both the yield stress and plastic viscosity decreased by 60.1% and 24.4%, respectively. Additionally, the attraction between particles, which encompasses electrostatic, van der Waals, and Lewis acid–base forces, decreased with a higher LS content. This suggests that the particle dispersion in AAS pastes improved as the LS content increased. However, there is currently limited research on the rheological properties of cementitious composites using LS. The impact of incorporating LS into concrete systems on the rheological performance of concrete warrants further investigation.

## 4. Mechanical Properties of Cementitious Composites with LS Incorporation

This section investigates results of the reported literature on the mechanical properties of LS-incorporated cementitious composites. It focuses on key parameters: the compressive strength, flexural strength, splitting tensile strength, and elastic modulus. Understanding how LS impacts these properties is essential for assessing the suitability of these composites in various construction applications. This section explores the relationship between LS content and these mechanical characteristics, thereby shedding light on the potential use of LS as a sustainable SCM to improve the performance of cementitious materials.

### 4.1. Compressive Strength

**Figure 8:**
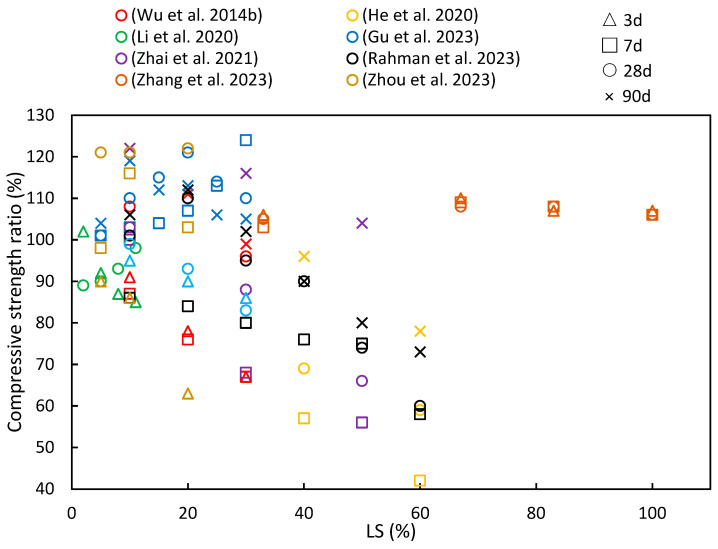
The compressive strength ratios of cementitious composites at various ages and levels of LS substitution [19,37,45,49,63,92,94,95].

Figure 8 summarizes the findings from numerous studies investigating the compressive strength ratios under different age and LS substitution conditions. A recurring pattern in most of the literature indicates that as the LS content increases, the compressive strength ratio initially rises before gradually declining, thereby implying an optimal LS content. He et al. [33] observed that an LS content exceeding 20% had an adverse effect on the compressive strength of concrete, while Luo et al. [102] noted that the geopolymer compressive strength significantly improved with LS-based geopolymer contents below 70%. Furthermore, as the curing period increases, there is a growing trend in compressive strength ratios. This phenomenon can be attributed to the late-stage pozzolanic activity of LS, as has been discussed in Section 2.2.

In the study by Zhou et al. [63], experiments were conducted to evaluate the reactivity of LS and SS as SCMs. The addition of 10% LS powder resulted in substantial hydration product formation at 28 days. LS exhibited a superior advantage over SS in enhancing the paste compressive strength. The pozzolanic reactions of LS and SS contributed to the development of the compressive strength at later stages. Additionally, in an effort to enhance the performance of ultra-high-performance concrete containing limestone powder (LP-UHPC), Zhang et al. [37] explored the partial replacement of LP with LS. The compressive strength of the LP-UHPC initially decreased and then increased with increasing LS content at all ages. Replacing 5% or 10% of the LP with LS led to improved compressive strength in the LP-UHPC across all age groups. However, the pozzolanic reaction of the LS exhibited limited the activity at the early stages of hydration. Rahman et al. [19] harnessed LS as an SCM to develop the pozzolanic activity. Inert and slowly reactive SCMs became pozzolanically active after 28 days, thereby making it convenient to assess inert, moderate, and highly reactive systems ranging from 0% to 60% in LS content in mortars. The results demonstrated that the 40% LS mortar achieved a 93% strength ratio compared to the mortar without LS within 28 days. Moreover, Zhai et al. [45] observed that when the LS powder content exceeded 30%, the early strength of the composite material decreased as the LS powder dosage increased. However, when the LS powder dosage was at 10%, the compressive strength at 28 days achieved a 21.5% increase compared to the sample group without LS. This improvement can be attributed to the increased reactivity of LS powder with age, thereby leading to a significant enhancement in the strength of the cured pastes over time. Gu et al. [95] employed LS as a replacement for SS in the preparation of cement mortar. They found that the 7-day compressive strength of the blended mortar increased as the LS/SS mass ratio increased. This enhancement was attributed to the high content of the reactive silica and alumina in the LS, along with a substantial amount of SO_4_^2−^, which provided early strength enhancement, as previously noted [34]. However, at 28 days, as the LS/SS mass ratio continued to increase, the compressive strength of the composite system showed an initial increase followed by a decrease. It can be concluded that when the LS content exceeds 20%, the reduced portlandite content in the matrix is insufficient to react with more LS to produce hydration products that can enhance the matrix strength. This conclusion is supported by findings from the studies of Wu et al. [92] and Li et al. [49]. He et al. [94] delved into the hydration mechanism of LS and concluded that with prolonged curing, the active and amorphous silicon dioxide and aluminum oxide within the LS slowly dissolved in the alkaline environment. These dissolved components then reacted with calcium hydroxide to form calcium silicate hydrate (C–S–H), thereby contributing to the increased later-age strengths of the samples. However, as the volume of the LS increased, its pozzolanic reaction became relatively slower. This occurs due to the dilution effect of cement, particularly at higher LS volumes; with the amount of cement being reduced in the mix, the calcium hydroxide content produced also reduces. The LS present in the mix reacts with the already available calcium hydroxide, and the excess LS material does not have any calcium hydroxide to prolong the pozzolanic reaction. Therefore, the excess LS slag particles are present as idle unreacted material, without contributing to the hydration characteristics of the cement blend.

### 4.2. Flexural Strength

**Figure 9:**
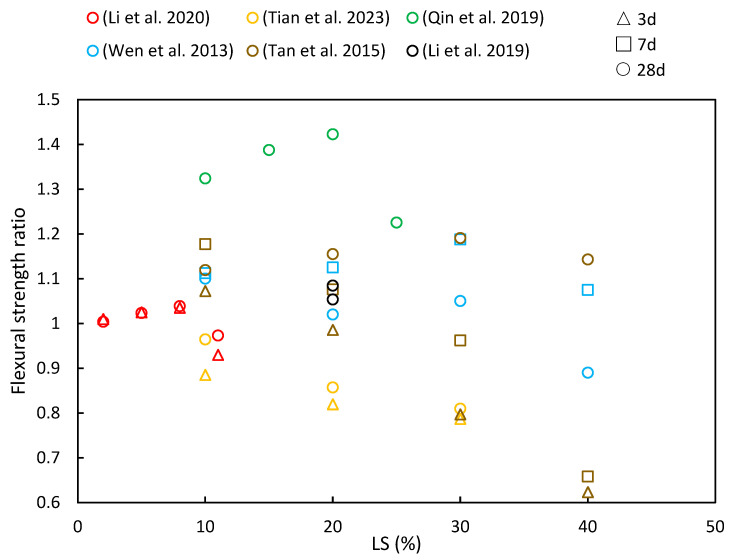
The flexural strength ratios of cementitious composites at various ages and LS substitution ratios [14,35,48,49,51,101].

Figure 9 provides a summary of the flexural strength ratios reported in various studies at different levels of LS incorporation and testing ages. The general trend of the flexural strength ratios closely resembles that of compressive strength, as has been discussed in Section 4.1. In most cases, the strength ratio tends to increase initially and then decrease with increasing LS content, with early-age strength ratios being slightly lower than later-age ratios. This behavior can be attributed to several factors: Firstly, when a small quantity of LS (e.g., 10% or 20%) is mixed with cement paste, it is initially considered inert, thereby not actively participating in the early-age hydration reactions. This results in a higher retention of water, thereby promoting the formation of gel and subsequently increasing the strength. Additionally, LS contains gypsum, which enhances the reactivity of silica and alumina, thereby leading to the production of more C–S–H and hydrated calcium aluminate.

Li et al. [49] demonstrated that the flexural strength of white reactive powder concrete increased with an added LS content of up to 8% at both 3 and 28 days but continued increases in the LS content led to reduced flexural strength. Their tests revealed that LS exhibited an impressive activity index of 82%, thereby surpassing that of fly ash and calcined gangue. Wen [35] also explored the flexural strength of green concrete by incorporating 10% LS and 10–40% LP. The flexural strength of the concrete showed an upward trend when the combined LS and LP amount was below 20%, thereby indicating that a moderate LS and LP had a positive effect on enhancing flexural strength. Additionally, Tan et al. [14] investigated the flexural strength in cementitious materials by replacing 10–50% of the cement with LS. Their results revealed that the early-stage flexural strength decreased significantly with higher LS content due to reduced cement content in the mixture. However, when the LS content remained at or below 20%, the compressive strength at 28 days exceeded that of OPC, although it decreased with an LS content beyond 20%. Moreover, in a study by Qin et al. [51] examining the effects of replacing cement with LS (10%, 15%, 20%, and, 25%), the maximum flexural strength was achieved with 20% LS across all the classes. These values exceeded the control specimens by significant margins, with increases of 17.8%, 33%, 46.1%, and 35.2% observed on the 28-day tests, respectively. The research concluded that higher LS replacement led to slower strength development in concrete samples.

Li et al. [48] conducted a study to investigate the flexural strength of 20% LS cement mortar under both wet curing and steam curing conditions. Their findings indicated that the addition of LS led to improved flexural strengths in mortars subjected to initial standard curing as well as steam curing. This enhancement was attributed to several factors, including the higher SO_3_ content in LS, which promotes the formation of more ettringite within the mortars. Additionally, the pozzolanic reaction of LS plays a role in consuming CH within the interfacial transition zone, thus further contributing to improved flexural strengths, particularly in the case of steam-cured LS samples [103]. However, Tian et al. [101] observed a different trend in modified AAS mortars following LS incorporation. They reported a decrease in the flexural strength with increasing LS content. Specifically, when the LS content reached 30 wt%, the flexural strength at 28 days decreased by 20.1%. This decline was attributed to the high degree of crystallization and resulting low reaction activity of the LS.

### 4.3. Splitting Tensile Strength and Elastic Modulus

**Figure 10:**
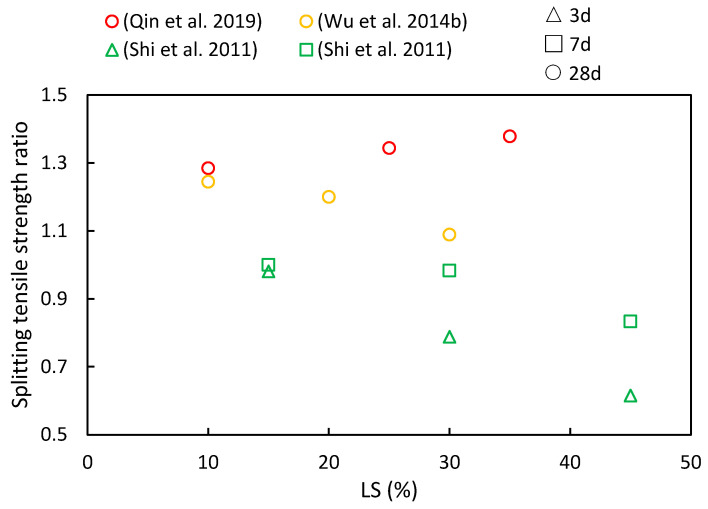
The splitting tensile strength ratios of cementitious composites at various ages and LS substitution ratios [51,53,92].

The splitting tensile strength ratios of cementitious composites at various ages and LS substitution ratios are depicted in Figure 10. It is evident from various literature sources that as the LS content increases, both increases and decreases in the splitting tensile strength ratios have been observed. However, with an increase in the curing period, the splitting tensile strength ratios were shown to consistently rise. At 28 days, the cementitious composites incorporating LS outperformed the control groups in the following studies, thereby highlighting the favorable long-term pozzolanic activity of LS. Qin et al. [51] employed LS derived from industrial waste to replace cement, thus aiming to enhance concrete’s mechanical properties. The results indicated that the splitting tensile strength initially increased and then decreased with a rising LS content, thereby reaching its peak improvement when the LS content substitution was at 25%. In addition, Wu et al. [92] observed that as the LS content ranged from 0% to 30%, the splitting strength values at 28 days exceeded those of the reference concrete. The maximum splitting strength was achieved at a 10% LS dosage, with a subsequent decline recorded as the LS content exceeded this threshold. Furthermore, Shi et al. [53] utilized LS as an SCM, thereby substituting 15–45% of the cement in high-performance concrete. The study revealed that the reduction in splitting tensile strength increased with a higher LS content at 7 and 14 days of testing.

**Figure 11:**
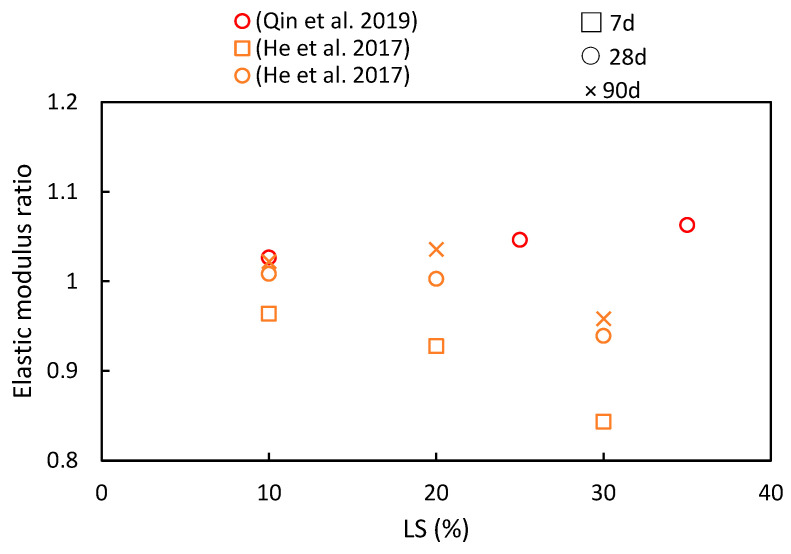
The elastic modulus ratios of cementitious composites at various ages and LS substitution ratios [50,51].

The elastic modulus serves not only as an indicator of concrete’s deformation characteristics, but it is also closely related to its compressive strength. Hence, variation in the elastic modulus ratio shares similarities with changes in the compressive strength, as indicated in Figure 11. Qin et al. [51] conducted an investigation into the impact of different LS substitution rates (0%, 10%, 25%, and 35%) on the elastic modulus ratios of concrete. As the LS content gradually increased, the peak elastic modulus was reached at a 35% LS content, thereby exhibiting an approximate 8.02% increase compared to the control group. This increase can be attributed to the secondary pozzolanic reaction of LS, which generates additional hydration products (C-S-H) to fill pores, thus consequently leading to an upward trend in the elastic modulus. However, exceeding this optimal point by adding excessive LS amounts can result in an overly pasty mixture, which is detrimental to the elastic modulus. Moreover, He et al. [50] experimentally assessed the influence of LS on the elastic modulus using specimens containing varying LS contents (0%, 10%, 20%, and 30% of the binder). It was observed that the LS had a noticeable impact on the elastic modulus development of the specimens. At 28 days, specimens with 10% or 20% LS exhibited higher elastic modulus ratios than the control specimen without LS. However, excessive paste content, resulting from an abundance of LS, hampers the enhancement of the concrete’s elastic modulus.

## 5. Durability of Cementitious Composites with LS Incorporation

This section probes into the essential durability properties of cementitious composites with the incorporation of LS. By investigating the chloride resistance, shrinkage, sulfate attack, and carbonation, we aim to gain insights into the long-term performance and resilience of these innovative materials. The interaction of LS with these durability aspects presents a critical dimension in understanding their practical applicability. Understanding these durability characteristics is pivotal in ensuring the sustainability and reliability of such composites in diverse environmental conditions.

### 5.1. Chloride Resistance

**Figure 12:**
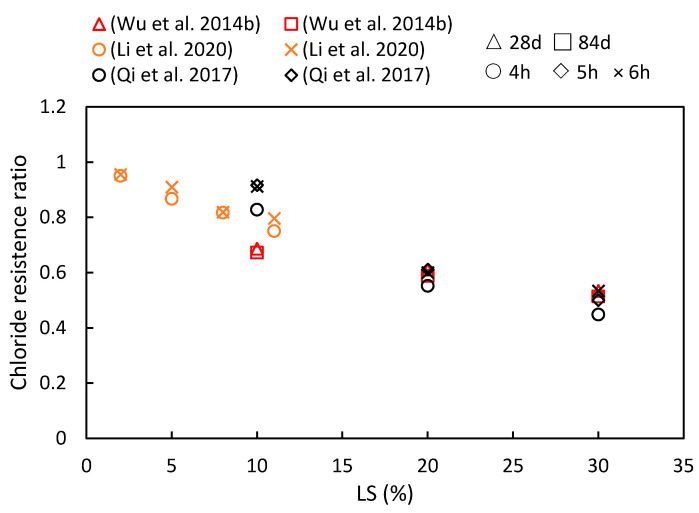
The chloride resistance ratios of cementitious composites at various ages and LS substitution ratios [49,56,92].

This study compiles the impacts of varying the LS content and the age of LS-based cementitious composites on chloride ion migration, as are summarized in Figure 12. The consistently observed chloride resistance ratio below one indicates that the LS enhanced the chloride ion migration resistance of cementitious systems. Furthermore, with an increase in the composite age, the resistance of the cementitious system to chloride ions has been shown to strengthen. Specific reasons for these findings are elucidated upon synthesizing the results from diverse studies. To begin with, Qi et al. [56] demonstrated a positive correlation between the LS content and the chloride penetration resistance. They reported a significant 43% reduction in the electric flux in concrete containing 30% LS compared to concrete without LS after 6 h of electrification. This improvement was attributed to the rapid secondary hydration of the LS and cement hydration products. This secondary hydration process transforms loosely bound Ca(OH)_2_ crystals, which tend to grow at the concrete interface, into a more compact gel-like layer, thereby reducing porosity and increasing chloride resistance. Additionally, Wu et al. [92] observed that the chloride ion diffusion coefficient gradually decreased with an increase in the total dosage of admixtures and extended curing time. The minimum chloride ion diffusion coefficient was achieved when the LS content was 30%, and the FA content was 20%. This reduction was due to the Al_2_O_3_ and SiO_2_ from the LS and FA engaging in secondary hydration reactions with the Ca(OH)_2_ in cement hydration. These reactions generated more hydrated aluminate and Friedel’s salt, thereby optimizing the structure of the interfacial zone and blocking penetration channels. Furthermore, Li et al. [49] measured the electric flux in samples at 28 days and found that different LS amounts enhanced the chloride penetration resistance. An 8% LS content led to an 18.9% reduction in the electric flux compared to the control group.

Therefore, the specific mechanism can be summarized as follows: The resistance of LS-based cementitious composites to chloride ion penetration is determined by two key factors—the inherent resistance to chloride ions and its physical or chemical capacity to bind with chloride ions. The introduction of LS plays a significant role in reducing concrete porosity and enhancing pore structure, thereby increasing the compactness of the interface between the aggregates and cement paste. Moreover, due to its highly active nature, LS undergoes a chemical reaction with the Ca(OH)_2_ and chloride ions in the cement matrix to form Friedel’s salt, thereby solidifying and preventing the migration of chloride ions as have been previously observed [104].

### 5.2. Shrinkage

**Figure 13:**
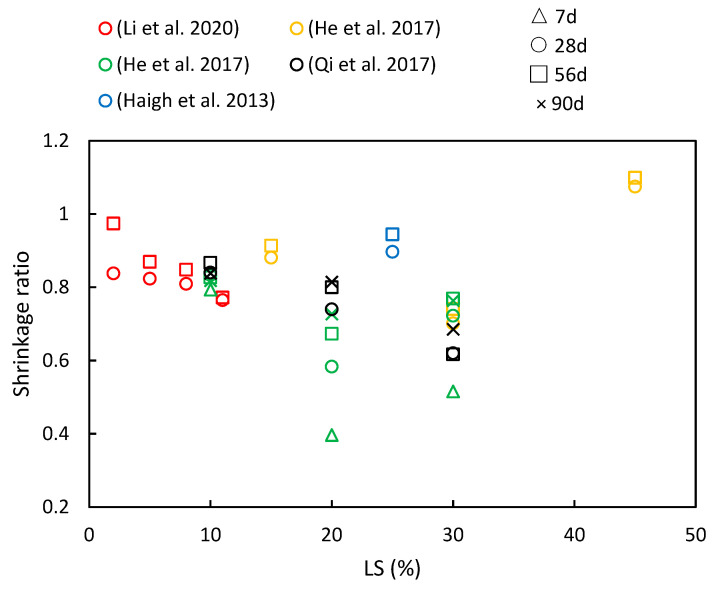
A compilation of shrinkage ratios in cementitious composites with varying LS contents and different ages [36,41,49,50,56].

Figure 13 presents a compilation of the shrinkage ratios in cementitious materials with varying LS contents and different ages, as have been reported in various studies. It is evident that increasing the LS content generally reduces the concrete’s shrinkage values. However, with the progression of age, the pattern of drying shrinkage exhibits various trends. The specific research findings are detailed below. Qi et al. [56] investigated concrete prepared by replacing cement with LS at ratios of 10%, 20%, and 30%. They found that the drying shrinkage remained steady, with a slow increase observed after 60 days. As the LS content increased, the drying shrinkage values decreased. For instance, compared to specimens without LS, the specimen with 30% LS exhibited a 23% reduction in dry shrinkage at 180 days. Furthermore, He et al. [36] explored the impact of LS on the drying shrinkage of concrete with manufactured sand at different ages. The results indicated that 15% and 30% LS effectively reduced the drying shrinkage of manufactured sand concrete, with a more pronounced effect at a higher LS content. The presence of smaller pores correlated with increased water retention, thereby leading to decreased drying shrinkage in manufactured sand concrete with an appropriate LS content. Similar conclusions were drawn by Li et al. [49]. Additionally, Haigh et al. [41] utilized 25% LS as a pozzolanic material in 25 MPa- and 40 MPa-grade concrete. Their study revealed that the 25 MPa LS concrete had 26.5% higher shrinkage than the FA concrete but was 2.3% lower than the control at 56 days. Moreover, the 40 MPa LS concrete exhibited the same shrinkage value as the FA concrete and was 12% lower than the control specimen. However, in the study conducted by He et al. [50], a contrary trend was observed concerning drying shrinkage. They presented data on the drying shrinkage strain of specimens with varying LS-to-binder ratios at different testing times. The results indicated that there is a limited upward effect of LS on the drying shrinkage strain, and among the three mixtures, the 20% LS appeared to be the most effective in reducing the drying shrinkage strain.

Through the literature review, it is evident that LS impacts concrete drying shrinkage through two primary mechanisms. On the one hand, the pozzolanic effect of LS enhances the formation of C–S–H secondary hydration products during the hydration process. These products fill the pores in the cement slurry, thereby leading to improvements in both the pore structure and interface structure within the concrete [25]. Additionally, being a porous mineral material [70], LS stores free water within its pore structure, which increases the water retention in the concrete and reduces water evaporation. This, in turn, effectively minimizes drying shrinkage [13].

### 5.3. Sulfate Attack and Carbonation

The investigation of sulfate attack and carbonation in cementitious composites with LS incorporation is vital for assessing their durability. Understanding how LS affects resistance to sulfate attack and carbonation is crucial for ensuring the long-term performance and sustainability of these materials when they are subjected to different exposure conditions.

Li et al. [48] conducted a study involving 20% LS mortar samples that were subjected to wet curing and steam curing, which was then followed by partial immersion in a 99% pure sodium sulfate solution for 720 days. Their findings indicate that, regardless of the initial curing conditions, the presence of LS enhances mortar properties in the face of sulfate attack. In addition to the role of leached LiAlSi_2_O_6_, the pozzolanic reaction of LS reduces the calcium hydroxide content within the mortar. Moreover, steam-cured LS mortar exhibited superior sulfate resistance compared to steam-cured PC mortar. Guangtai et al. [105] conducted experiments to investigate the mechanical properties of a novel concrete subjected to sulfate attack. They utilized a 5% mass-fraction sulfate solution for accelerated erosion tests on 11 sets of polypropylene fiber-reinforced lithium slag concrete (PLiC) specimens and eight large eccentrically loaded PLiC columns. The results revealed that the addition of LS improved the sulfate resistance of the polypropylene fiber-reinforced concrete columns. Additionally, in damaged members, the fractal dimensions of the surface cracks exhibited an increasing trend with sulfate erosion duration. Lastly, in a study by Qin et al. [57], two groups with LS substitution rates of 20% and 25% (LS20 and LS25) were analyzed for microstructural changes to explore the damage mechanism under sulfate erosion combined with freeze–thaw cycles. The increased SO_4_^2−^ concentration in the LS25 indicated the presence of more expansive products, such as ettringite and flaky gypsum, which accelerated corrosion and led to fundamental specimen deterioration. The internal structural damage was more pronounced in the LS20 compared to the LS25, despite the LS25 having a lower macroscopic damage index.

Research on the carbonation of cementitious composites with LS incorporation remains limited at present. Qi et al. [56] conducted a study to investigate the influence of LS on the carbonation resistance of concrete. They examined the carbonation depth at various LS ratios and concrete ages. The results indicated that with a higher LS content and longer concrete curing periods, the carbonation depth increased. This phenomenon can be attributed to LS replacing cement and undergoing secondary hydration with cement hydration products, thereby resulting in decreased alkalinity within the concrete. As a consequence, the neutralization resistance decreases during CO_2_ infiltration, thereby leading to a gradual reduction in the carbonation resistance of the concrete as the LS content increases.

## 6. Chemical and Microstructural Investigations of Cementitious Composites with LS Incorporation

This section delves into the microscopic analysis of LS-based cementitious composites, thus focusing on critical aspects such as hydration heat, pore structure, XRD, SEM, FTIR, and TG. Through these analytical tools, we aim to unravel the intricate details of the composite’s microstructure and chemical composition. Hydration heat reveals the impact of LS on the setting time and the ultimate strength development of cement blends. Pore structure assessments unveil the impact of LS on porosity, while XRD elucidates the crystalline phases present. SEM provides high-resolution images, thereby offering insights into surface morphology. FTIR and TG shed light on chemical interactions and thermal behavior. This comprehensive analysis serves as a foundational exploration of cementitious composites with LS incorporation at the microscopic level.

### 6.1. Hydration Heat

The evolution of hydration heat plays a fundamental role in the initial setting, hardening, and ultimate strength development of cement blends. The effective control of hydration heat is imperative to ensure the overall quality and durability of concrete structures. Upon contact with water, cementitious materials undergo a series of chemical reactions, thereby resulting in a distinct hydration heat evolution curve that typically encompasses several stages, including the initial period, induction period, acceleration period, deceleration period, and retardation period [106,107,108].

He et al. [109] noted that that the incorporation of 20 wt% LS into a composite binder led to a notable reduction in the evolution of hydration heat and a significant delay in the induction period when compared to pure cement paste. This phenomenon can be attributed to the reduced clinker content resulting from the substitution of Portland cement with LS. In addition, during the early stages of hydration, LS was found to exhibit inert behavior and exhibited limited participation in the initial hydration reactions. Moreover, Zhai et al. [45] conducted a study revealing that the induction period of cement blends, incorporating LS powder, fell within the range of 1.8 to 4.1 h. Notably, when the LS powder content reached 50%, the induction period of the blended cement extended by 2.26 h compared to that of pure cement. Furthermore, the second exothermic peak value was only 51.5% of that observed in pure cement paste. The extension of the induction period underscores the retarding influence of LS powder on the initial hydration of the cement. In a study by Rahman et al. [70], the heat flow analysis of LS cement pastes ranging from 0% to 60% in LS content provided crucial insights into the dormant, primary, and secondary hydration peaks. Despite the higher concentration of LS diluting the clinker, the induction period was slightly prolonged, which is the same as the conclusions of References [45,109]. However, an intriguing observation was the substantial reduction in the initial setting time for pastes containing 40% and 60% LS in comparison to the control. This phenomenon was attributed to the significant aluminum oxide content within the LS, which, at higher replacement levels, rendered the pastes susceptible to flash setting due to the rapid hydration nature of the alumina, which is akin to the well-understood rapid setting of the C_3_A phase in cement. Lastly, Tan et al. [88] noted that nano-LS accelerated the hydration of SAC. Remarkably, a dosage of 4.0% even eliminated the induction period entirely. The acceleration of hydration was attributed to the nanoparticles within the nano-LS slurry, which acted as highly effective nucleation seeds, thereby expediting the formation of hydration products. Additionally, the enhanced dissolution of lithium salt during the wet milling process facilitated the precipitation of the aluminum phase, thus further expediting SAC hydration.

### 6.2. Pore Structure

The construction of pores, specifically porosity and pore size distribution, significantly influences concrete properties [110]. He et al. [50] examined the impact of LS on pore size distribution at 7 days and 90 days, which was measured via MIP. As the age increased, the pore size and volume decreased. The introduction of LS initially increased the porosity of larger pores. However, including 10% and 20% LS in the binder refined the pore structure in later stages. In addition, He et al. [94] observed that replacing 40% of their cement sample with LS reduced the porosity in the later curing stages, thereby correlating with improved mechanical properties. However, the use of 60% LS increased the total porosity, particularly for pores exceeding 100 nm in diameter. This was attributed to the incomplete stimulation of the LS pozzolanic reaction due to the limited calcium hydroxide amount generated during Portland cement hydration. Wang et al. [61] found that introducing C-A-S-H seeds with a Ca/Si ratio of 1.5 significantly reduced the volume proportion of harmful large pores in LS-blended cement paste, thereby refining the pore structure. Additionally, Zhang et al. [37] demonstrated that LS addition reduced UHPC porosity at 28 days due to pozzolanic reactions, the filler effect, and reactions between the LP and LS aluminate phases. Lastly, Li et al. [48] replaced 20% of their cement sample with LS and assessed changes in the pore structure under standard and steam curing. Under standard curing, the LS reduced the porosity and capillary pore volume. Similarly, under steam curing, LS incorporation promoted gel pore formation and reduced the capillary pores due to the presence of gypsum and carbonate in the LS, which accelerated cement hydration.

Following the literature review, three reasons can be identified to explain these phenomena. Firstly, the filling effect is significant. The fine-grained nature of LS enhances particle packing, while the fine LS particles serve as pore blockers, thereby reducing pore interconnectivity and effectively lowering porosities. Secondly, the pozzolanic reaction of LS plays a crucial role. LS reacts with CH to generate additional hydration products, thus refining large pores and bridging the gap between pastes and aggregates. Lastly, LS provides nucleation sites, thereby promoting the preferential production and development of hydration products in these locations.

### 6.3. XRD Analysis

**Figure 14:**
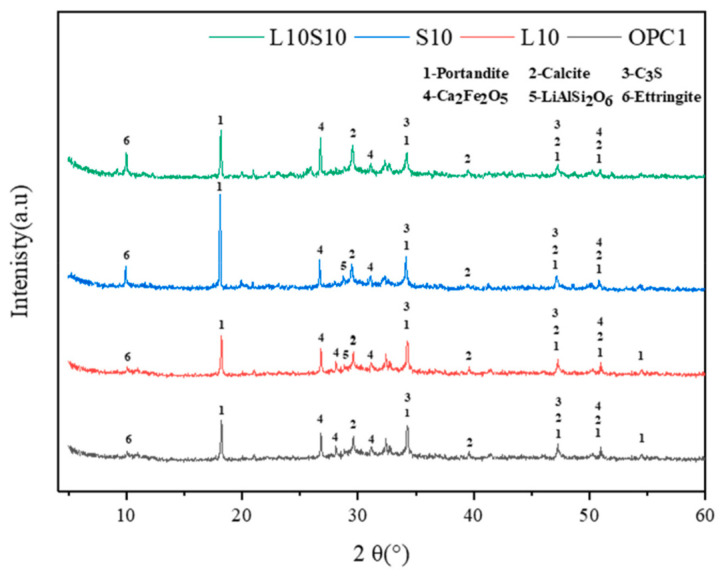
XRD patterns of cement pastes [63].

XRD analysis provides insights into the crystalline phases within samples. Zhou et al. [63] generated XRD patterns for specimens containing LS and SS following standard curing for 7 days, as are illustrated in Figure 14. The primary mineral composition of the cementitious materials was identified as portlandite, calcite, C_3_S, Ca_2_Fe_2_O_5_, LiAlSi_2_O_6_, and ettringite. Notably, the presence of LiAlSi_2_O_6_ was detected by a prominent peak between 2θ = 25–30°, which was observed in the samples with the 10% LS replacement due to the introduction of spodumene (LiAlSi_2_O_6_) from the LS. The diffraction peak intensity of the lithium pyroxene was low, thereby indicating a predominance of amorphous Al_2_O_3_ and SiO_2_ within it [111]. Furthermore, because spodumene was an inert component in the raw LS material and exhibited limited participation in the hydration process, traces of it persisted in the LS-mixed samples.

**Figure 15:**
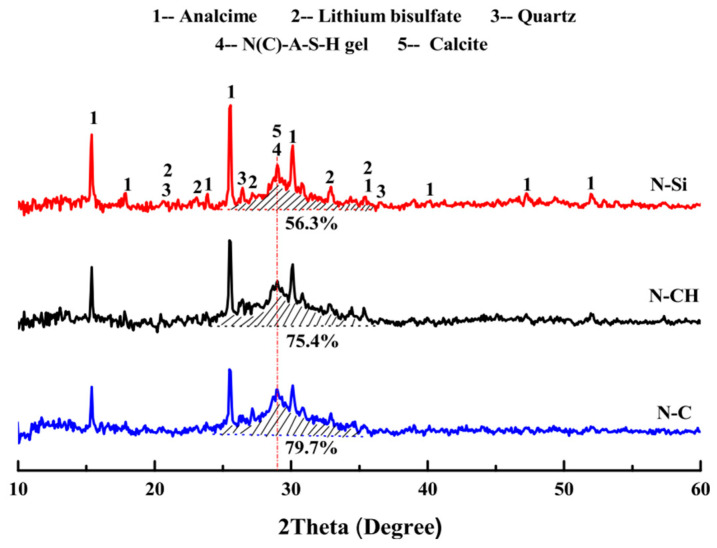
XRD patterns of LS-based geopolymers cured for 28 days [24].

In the study conducted by Luo et al. [24], LS was employed in the preparation of one-part geopolymers using three hybrid solid activators: NaOH + Na_2_SiO_3_, NaOH + Ca(OH)_2_, and NaOH + CaCO_3_. Figure 15 illustrates the XRD patterns of LS-based geopolymers cured for 28 days. The primary crystalline phases identified in the geopolymers included analcite, calcite, and lithium bisulfate. It was observed that the proportion of the integral diffraction peak area attributed to the N(C)-A–S–H gel in the N-CH and N–C samples was greater than that in the N-Si sample. This finding suggests that the NaOH + Ca(OH)_2_ and NaOH + CaCO_3_ hybrid activators exhibited a more effective activation effect on LS-based geopolymers compared to NaOH + Na_2_SiO_3_ hybrid activators.

Furthermore, Guo et al. [16] synthesized ternary AAMs by combining LS, MK, and GGBS. The XRD patterns of the AAM pastes at 28 days revealed the presence of key phases, including quartz, hydrotalcite, calcite, hydrogen aluminum silicate, C-(A)-S-H, and gypsum. LS and MK contributed aluminum and silicon sources in an alkaline environment, thereby facilitating AAM polymerization due to the presence of the H(AlSi_2_)O_6_ phase and glass phase. Additionally, variations in the contents of CaO, Al_2_O_3_, and SiO_2_ in the LS and MK amounts, as the LS replaced the MK, led to corresponding changes in the proportions of these three oxides in the precursor, thereby potentially impacting the formation of the C-(A)-S-H phase.

### 6.4. SEM Analysis

**Figure 16:**
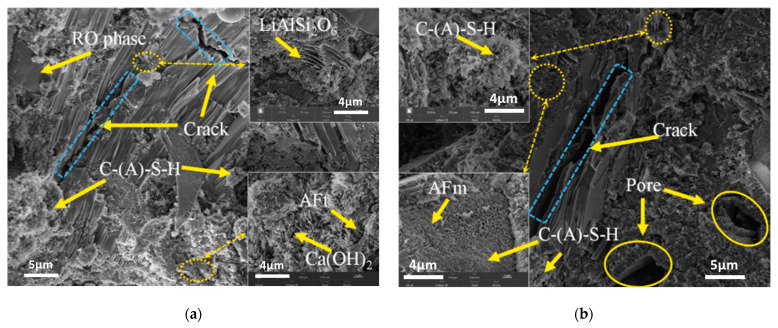
SEM images of (**a**) 10SS-20LS and (**b**) 5SS-25LS at 28 d [95].

Investigating the microstructure of cementitious materials aids in explaining the mechanical strength of the matrix, as well as the extents and modes of reaction of different raw materials, thus elucidating the reaction mechanism of LS within the matrix. In the study conducted by Gu et al. [95], it was found that in the C-LS-SS cementitious system that SS and LS replaced 10% and 20% of the cement, respectively (10SS-20LS), LiAlSi_2_O_6_ and RO phases were also observed, and the layered spodumene (LiAlSi_2_O_6_) in the LS underwent partial leaching, as is depicted in Figure 16a. With an increase in the LS content, there was a significant consumption of portlandite, thereby leading to a reduction in the degree of LS reaction. In addition, the addition of SS had a detrimental impact on the pore structure of the pure cement system, thereby increasing the number of pores and fractures, which is consistent with previous findings [112]. However, in this study, the addition of SS-LS improved the pore structure of the composite system, thereby resulting in a denser surface. Notably, evident voids and large cracks were observed in the hydration products of 5SS-25LS (SS and LS replacing 5% and 25% of the cement, respectively) shown in Figure 16b. These results indicate the formation of delayed ettringite due to the high SO_3_ content in the LS [113], which subsequently led to the development of microcracks.

**Figure 17:**
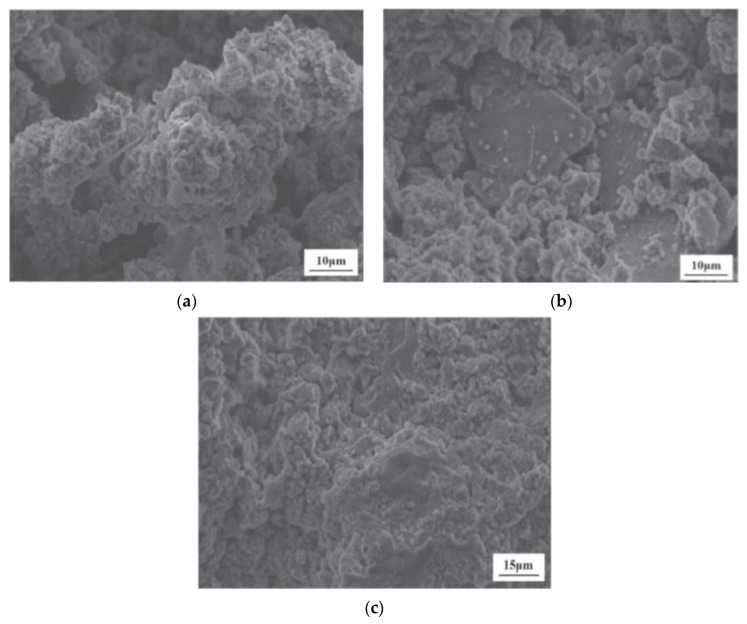
SEM images of geopolymers at the age of 28 d: (**a**) G0, (**b**) G20, and (**c**) G40 [102].

Luo et al. [102] introduced a highly efficient approach for the large-scale utilization of LS in the synthesis of LS-based geopolymers using a one-part mixing method. SEM images of LS-based geopolymers at 28 days are presented. As is illustrated in Figure 17a, the surface of G0 (without GGBS) primarily consisted of numerous unhydrated LS particles, with few hydrates forming on their surfaces. This suggests a low degree of LS hydration. Consequently, there were insufficient hydration products to fill the pores within the LS and the gaps between the LS particles. In contrast, as are depicted in Figure 17b, some hydration products of the LS and GGBS were visible on the surface of the geopolymer with the GGBS replacing 20% of the cement (G20) content. Nevertheless, the quantity of hydration products generated was inadequate to completely occupy the spaces between the LS and GGBS particles and promote cementation. Upon reaching a GGBS content of 40% (G40), substantial hydration products were observed on the surfaces of the G40 geopolymer particles shown in Figure 17c, thereby effectively filling the voids between the LS and GGBS particles and enhancing the compactness of the geopolymer structure.

**Figure 18:**
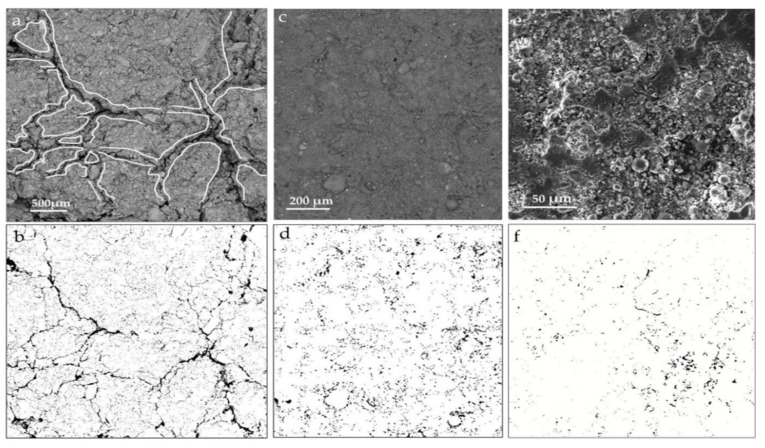
Calculation of void area from SEM micrographs of LS-FA geopolymer specimens. (**a**,**b**): 100LS0FA; (**c**,**d**): 50LS50FA; (**e**,**f**): 0LS100FA. Note: the number represents the relative proportion of LS and FA [23].

Interestingly, Javed et al. [23] employed SEM images to calculate the internal porosity of geopolymers. The electron micrographs in Figure 18 illustrate the percentage voids in the LS geopolymer containing FA. Even at lower magnifications, cracks and voids were evident in the 100% LS geopolymer paste, thereby indicating higher porosity. The percentage void areas for the 100LS0FA, 50LS50FA, and 0LS100FA mixtures (the number before the abbreviations represents the relative proportion of LS and FA) were 7.29%, 4.76%, and 1.20%, respectively. The incorporation of 50% FA in the LS geopolymer led to a reduction in the crack formation, which was attributed to sulfate dilution in the pore solution and decreased cracking. Therefore, porosity is closely associated with the sulfate ions in geopolymer pastes.

Based on the findings from these studies, it can be concluded that the moderate addition of LS, especially when mixed with other SCMs, is beneficial for achieving a denser microstructure in both cementitious and geopolymer systems. However, excessive LS addition can increase the porosity of the binder materials, thereby reducing their mechanical strength. Although LS exhibits lower early-stage hydration reactivity, it actively participates in the hydration process in later stages, thereby contributing to a more compact matrix.

### 6.5. FTIR and TG Analysis

**Figure 19:**
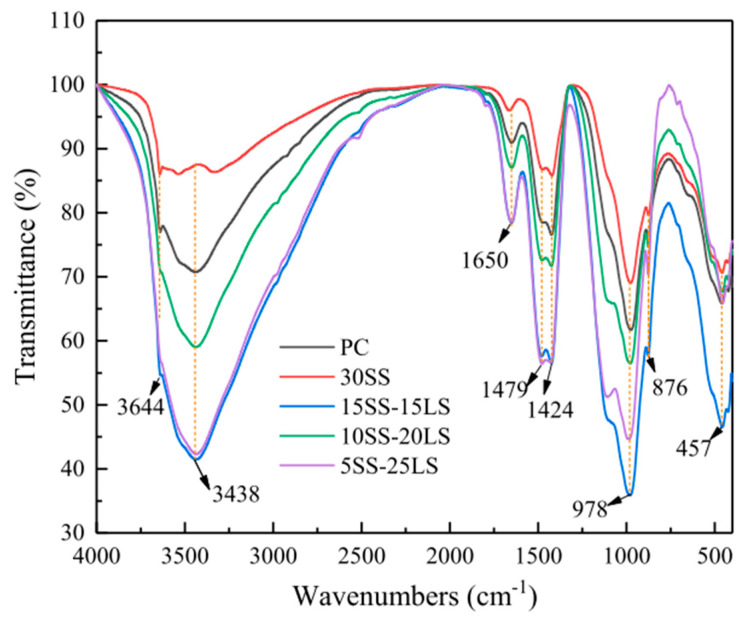
FTIR spectra of pastes at 28 d [95].

**Figure 20:**
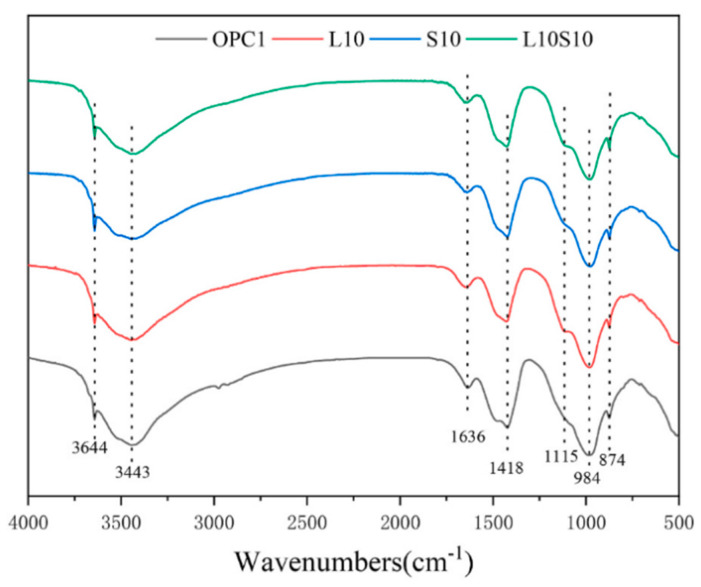
FTIR spectra for cement samples at 7 d [63].

SCMs were produced by blending SS and LS, and their synergistic effect was investigated by Gu et al. [95]. The characterization of the hydration products was conducted using FTIR, as is depicted in Figure 19. The faint peaks at 3644 cm^−1^ originated from the bending vibration of the OH groups in portlandite [114]. These spectral peaks associated with portlandite exhibited a reduction in intensity with an increasing LS content. Moreover, the absorption peaks at approximately 978 cm^−1^ were linked to the asymmetric stretching vibrations of Si–O–Si (Si–O–Al) related to C-S-H gels [115]. Among the composite systems, the 10SS-20LS group exhibited the highest peak strength, thus slightly surpassing that of the PC and signifying that the SS-LS demonstrated more pronounced pozzolanic activity at suitable proportions. In experiments conducted by Zhou et al. [63] to assess the reactivity of LS and SS as SCMs, variations in the peak depth were observed in each sample, as are shown in Figure 20. Notably, changes in the depths of the peaks corresponding to the hydroxyl groups in the Ca(OH)_2_ and the Si–O bonds in the Si–O tetrahedra were noted with the introduction of LS and SS. The peak depths in the reference group (OPC1) were lower than those in the L10 and S10 groups but higher than those in the L10S10 group. This observation suggests that the addition of LS or SS increased the degree of cement hydration. The combination of LS and SS (L10S10) reduced the extent of cement hydration, thereby aligning with the compressive strength results at 7 days.

**Figure 21:**
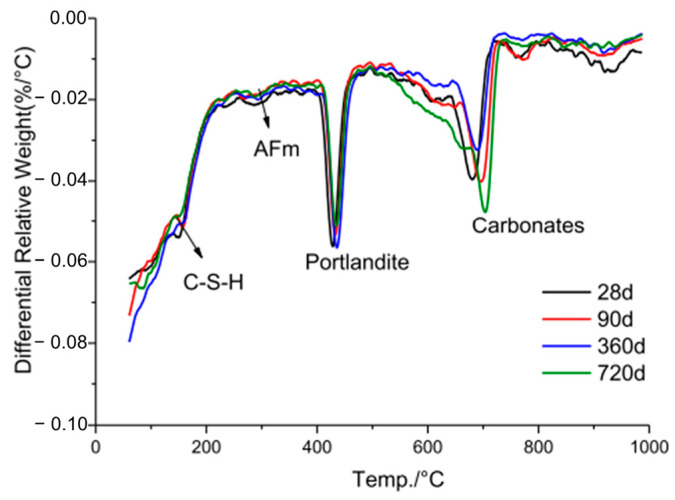
Hydration products of LS mortar cured under 80 °C steam for 7 h at different ages [48].

Tan et al. [30] compared the TG profiles of 4% micro-LRR with control specimens, thereby revealing that the LS samples had lower levels of initial hydration products and portlandite consumption than the control samples. Zhang et al. [34] conducted a TG comparison between 30% LS (control) and 30% LS plus 0.06% TIPA-containing mortar specimens, and their results were consistent with those of Tan et al. [30]. Li et al. [48] conducted a comparative TG analysis of mortar samples containing 20% LS, which were subjected to both normal and heat curing conditions, as is shown in Figure 21. The results revealed that the mortar cured under 80 °C steam for 7 h exhibited lower mass loss than the normally cured sample. This suggests that the formation of the AFt and C-S-H phases in the steam-cured sample was less extensive than in the normally cured sample. The elevated steam curing temperature led to the decomposition of the AFt and C-S-H products. Additionally, the mass loss of the steam-cured samples between 400–500 °C was higher than that of the normal samples. While portlandite consumption was higher, the increased loss of initial hydration products resulted in inconsistent strength development. Li et al. [49] conducted TG tests, thereby yielding results similar to those of Li et al. [48]. Mortar samples containing 11% LS exhibited higher portlandite consumption but reduced levels of the initial hydration products. Finally, He et al. [26] assessed the mass loss of control specimens and specimens containing 24% LS plus 6% NaOH. The LS-containing sample exhibited lower levels of initial hydration products and portlandite consumption than the control sample, thereby resulting in a 3.3% reduction in the unconfined compressive strength of the LS-containing backfill specimen at 28 days compared to the control specimen. In the study conducted by Zhang et al. [37], a UHPC was prepared by substituting 30% of the cement content with varying proportions of LS and LP, and a quantitative investigation of the pozzolanic activity of the LS was conducted using TG analysis. It was observed that the UHPC sample containing 30% LS exhibited the highest bound water content (14.4%). However, the bound water content decreased to 12.6% as the content of the LP increased to 30% of the cement. This phenomenon can be attributed to the enhanced pozzolanic reaction of LS at the later stages of hydration, thereby resulting in the generation of more hydration products within the LS-UHPC and increasing the bound water content. Additionally, LS exhibits pozzolanic activity and reacts with Ca(OH)_2_, thereby leading to a reduction in the Ca(OH)_2_ content in the UHPC. Quantitative analysis from TG curves indicated that as the LP progressively replaced the LS from 0% to 100%, the Ca(OH)_2_ content in the UHPC increased from 6.1% to 7.6%, respectively.

Based on the current state of the research, the microscale testing methods employed in this section, such as XRD, SEM, and TG, primarily serve the purpose of providing qualitative investigations into the pozzolanic reactivity of LS. However, it is noteworthy that there is little research on the quantitative studies of pozzolanic activity using these microscopic methods. A quantitative assessment of the pozzolanic reactivity of LS in cementitious materials has been reported solely by Zhang et al. [37], who utilized TG to calculate the bound water content and calcium hydroxide content in LS-based concrete as a means of assessing its pozzolanic reactivity. Furthermore, through a comprehensive literature review, it was revealed that quantitative investigations into the pozzolanic reactivity of LS have been conducted by Rahman et al. [19] using nonmicroscale testing methods such as the Frattini test, strength activity index (SAI), and R^3^ test. The findings indicated that when 40% LS was employed as an SCM, it reacted with 79% of the CaO content in the cement mix, thereby resulting in a 93% SAI at 28 days and generating 53.1 J/g of SCM hydration heat with portlandite at 7 days. However, it is noteworthy that the utilization of 20% LS as an SCM yielded the maximum SAI, while higher percentages (50–60%) resulted in diminished strength and hydration heat. It is well known that the pozzolanic reactivity of LS is closely intertwined with its potential application as an SCM into cement blends. Consequently, there is a need for further in-depth quantitative investigations into the pozzolanic reactivity of LS.

## 7. Cost, Energy, and Carbon Emission Comparisons

The ineffective disposal of LS can pose a serious environmental threat due to the leaching of fluorine and sulfate ions, which can lead to pollution of both land and water. Therefore, it is imperative to explore methods for the disposal and efficient utilization of LS to support the sustainable development of the lithium carbonate and construction materials industries. In order to facilitate the widespread application of LS in the construction materials sector, it is equally important to assess its commercial viability. Hence, this study presents a comparative analysis of LS with common cementitious composites in terms of cost, embodied CO_2_, and embodied energy, as is summarized in Table 4.

It is evident that LS has the lowest cost (10 USD/t) compared to other construction materials. Not only does LS exhibit similar pozzolanic activity to FA, but it also comes at just one-fifth of the price of FA. This makes LS an economically attractive option for concrete or geopolymer production, thereby significantly reducing manufacturing costs. However, it is worth noting that Ali et al. [5] reported the cost index values for a novel LS-based geopolymer, and it is interesting to observe that the geopolymer with a higher LS content resulted in higher cost index values. This is attributed to the very low strength of the binders prepared from higher LS contents. Furthermore, it is important to mention that the cost index values for all geopolymer mixes are higher than the 1.75 USD/m^3^·MPa cost index value reported for a previously studied OPC mix [116]. The higher cost of geopolymer binders can be attributed to the expensive sodium silicate and recent price increases in industrial waste materials (FA and slag) due to government-imposed limitations on coal and steel production. Additionally, in the study conducted by Guo et al. [16], the cost index of alkali-activated material pastes containing LS showed varying degrees of reduction compared to the control sample. The cost index of the paste without LS was 4.68 USD/(m^3^·MPa). As the ratio of LS replacing metakaolin increased from 25% to 75%, the cost indexes decreased to 2.94 USD/(m^3^·MPa), 2.40 USD/(m^3^·MPa), and 2.23 USD/(m^3^·MPa), respectively. This reduction can be attributed to the fact that the cost of LS (10 USD/t) is significantly lower than that of MK (220 USD/t).

Table 4 also reveals that the embodied CO_2_ and embodied energy of LS are comparable to GGBS, slightly higher than those of FA and SF, and significantly lower than those of MK, LP, and cement. Therefore, LS exhibits the potential for solid waste recycling and sustainable development. Das et al. [117] reported an embodied CO_2_ index of approximately 18 kg/m^3^·MPa for OPC. Ali et al. [5] found that the embodied CO_2_ index values of geopolymers with 70% and 60% LS are 17% and 39% lower, respectively, than that of OPC, thereby showcasing its superior environmental performance and strength characteristics over OPC. The lower index values for geopolymers with 80%, 90%, and 100% LS are due to the lower compressive strength values of these mixes, not the actual embodied CO_2_ values, which are significantly lower than OPC for all the compositions of geopolymer binders. Furthermore, the embodied CO_2_ index was used to assess the sustainability of the developed UHPC [37]. LP, by replacing with either 5% or 10% LS, reduced the carbon index of LS-UHPC. However, further increases in the LP content significantly compromised the compressive strength of the UHPC. LP20 and LP30, in particular, increased the carbon emissions of LS-UHPC, even when considering performance. On the other hand, LP5 and LP10 were able to recycle a substantial amount of LS while ensuring excellent UHPC performance. Therefore, adding 5% or 10% LP not only enhanced the performance of LS-UHPC but also offered environmental and sustainable development advantages.

Regarding the energy index, Guo et al. [16] demonstrated that the energy consumption of LS (2230 MJ/t) is slightly lower than that of MK (2500 MJ/t), thereby implying that replacing MK with LS can reduce energy consumption to some extent [16]. In AAM pastes with LS replacing MK, the higher strength of LS-containing AAM compared to AAM without LS does not provide a clear advantage in terms of the energy index. However, this substitution strategy holds strong potential for improving the construction environment and conserving natural resources. Ultimately, based on a comprehensive analysis of workability, strength, reaction degree, microstructure, cost, and energy consumption, this work recommends replacing 25% to 50% of MK content with LS in the AAM GBFS-MK-LS ternary system.

**Table 4:** A comparative analysis of LS with common cementitious composites in terms of cost, embodied CO_2_, and embodied energy.

Materials	Cost (USD/t)	Embodied CO_2_ (kg/t)	Embodied Energy (MJ/t)
LS	10 [5]	67 [118]	2230 [22]
GGBS	100 [119]	67 [118]	1590 [119]
MK	220 [120]	400 [121]	2500 [120]
FA	50 [122]	8 [123]	833 [121]
SF	200 [124]	14 [125]	100 [126]
C	467.5 [127]	900 [128]	5000 [129]
LP	150 [130]	75 [118]	350 [121]

## 8. Conclusions

This study provides a comprehensive overview encompassing the physiochemical and microscopic properties of LS, fresh state properties, mechanical characteristics, durability, and the microscopic analysis of cementitious composites incorporating LS. Additionally, it includes cost, energy, and carbon emission comparisons between LS and other cementitious materials. The following conclusions can be drawn from this extensive review:

(1) The PSD of LS closely resembles that of FA and GGBS. This similarity suggests that LS can exhibit similar effects related to densification and nucleation when integrated into concrete, thus resembling the behavior of FA and other SCMs. Mechanical treatment of LS enhances the dissolution of aluminum, lithium, and silicon in LS, thereby expediting early hydration in LS–cement systems.
(2) LS exhibits variations in SiO_2_ + Al_2_O_3_ and Ca/(Si + Al) within the ranges of 70.29–80.77% and 0.02–0.14%, respectively. This composition aligns LS with FA, which is characterized by high SiO_2_ and Al_2_O_3_ contents and a low CaO content. This similarity categorizes LS as a low-calcium precursor with chemical reactivity akin to that of FA.
(3) In most of the literature examined, an increase in LS content was shown to lead to a reduction in the initial and final setting times of LS–cement and LS–geopolymer systems. Moreover, the studies determined that flowability decreased with an increase in LS content due to its irregular shape, strong water absorption characteristics, and elevated formation of AFt in the initial stages of hydration.
(4) A recurring trend in most of the reviewed literature indicates that as LS content increases, the compressive strength, flexural strength, and splitting tensile strength ratios initially increase, with diminishing returns beyond a 30% threshold. This suggests an optimal LS content for achieving favorable mechanical properties. Additionally, with longer curing periods, there is a noticeable upward trend in the compressive strength, flexural strength, and splitting tensile strength ratios.
(5) LS plays a crucial role in enhancing chloride ion migration resistance and reducing shrinkage in cementitious systems. Furthermore, as the composite ages, the resistance of the cementitious system to chloride ions becomes more robust. However, the behavior of drying shrinkage exhibits various trends.
(6) The mechanisms through which LS operates within cementitious composites can be classified into three main categories. Firstly, there is the filling effect: the fine-grained nature of LS improves particle packing, and its fine particles act as pore blockers, thereby reducing interconnectivity between pores and effectively lowering porosity. Secondly, there is the pozzolanic effect: LS reacts with calcium hydroxide to generate additional hydration products, thereby refining large pores and bridging the gap between the paste and aggregates. Thirdly, there is the nucleation effect: LS provides nucleation sites, thereby promoting the preferential production and development of hydration products in these specific locations.
(7) LS not only exhibits similar pozzolanic activity to FA, but it also comes at just one-fifth of the price of FA. This makes LS an economically attractive option for concrete or geopolymer production, thereby significantly reducing manufacturing costs. Moreover, the embodied CO_2_ and embodied energy of LS are comparable to GGBS, slightly higher than those of FA and SF, and significantly lower than those of MK, LP, and cement. Therefore, LS exhibits the potential for solid waste recycling and sustainable development.

## 9. Outlook

Despite extensive research on LS, there remain several performance aspects and application areas warranting further in-depth investigation:

(1) Current research on the grinding and chemical treatment of LS is limited. Further exploration is needed to enhance its utilization efficiency through physical and chemical modifications.
(2) More research is required to understand the tensile properties of cementitious composites incorporating LS and their durability evolution in specific environments, such as freeze–thaw cycles and exposure to coupled acid–base and salt conditions.
(3) Further exploration into the performance of LS in high-performance concrete, such as UHPC and engineered cementitious composites, is warranted.
(4) The rheological properties of LS when incorporated into cement pastes and its subsequent performance in 3D printing applications deserve closer attention.
(5) Investigation into the hydration mechanisms of LS when used in specialized cements, such as SAC and limestone calcined clay cement, requires further research.
(6) The current quantitative research on the pozzolanic reactivity of LS is limited. A thorough assessment of the pozzolanic reactivity of LS is needed to confirm its suitability as an SCM in cement blends.

Addressing these research gaps will contribute to a more comprehensive understanding of LS and its potential applications in the field of cementitious materials. LS offers varied prospects in construction, thereby serving as a supplementary element in concrete to boost durability and sustainability. It spans across structural applications and innovative material development, thereby enhancing performance, reducing waste, and possibly cutting project expenses. Engineers can adjust the workability, mechanical properties, and durability aligning with regulations, and they can seek ecofriendly solutions for resilient, cost-effective construction methods.

## Figures and Tables

**Table 1:** The raw material system of LS-based cementitious composites.

System	Specific Combination	References
Binary	LS-C	[29,30,31]
Ternary	LS-FA-C	[25,32]
	LS-SF-C	[33]
	LS-TIPA-C	[34]
	LS-LP-C	[35,36]
Quaternary	LS-GGBS-FA-C	[32]
	LS-LP-SF-C	[37]
	LS-PS-SS-C	[38]
	LS-PCE-TEA-C	[39]

C: cement; FA: fly ash; SF: silica fume; TIPA: triisopropanolamine; LP: limestone powder; SS: steel slag; GGBS: ground granulated blast slag; PS: phosphate slag; PCE: polycarboxylate; TEA: triethanolamine.

**Table 2:** Particle size distribution of LS in different studies.

Reference	D10 (µm)	D50 (µm)	D90 (µm)
[38]	1.56	13.00	81.00
[45]	2.90	7.10	42.80
[39]	-	11.80	-
[61]	-	30.39	-
[37]	0.84	6.24	28.00
[63]	2.74	25.26	83.65
[43]	-	4.53	-
[19]	-	38.00	-
Average	2.01	17.04	58.86

**Table 3:** Chemical compositions of LS (wt%).

Reference	SiO_2_	Al_2_O_3_	Fe_2_O_3_	SO_3_	CaO	MgO	K_2_O	Na_2_O	LOI
[34]	52.21	20.60	0.84	9.18	4.63	0.16	0.26	0.33	11.39
[42]	40.33	34.51	2.25	-	18.47	0.05	-	-	-
[22]	54.53	21.08	1.45	5.62	7.54	0.58	0.89	0.72	6.76
[27]	48.97	21.32	1.07	16.2	8.26	0.19			3.37
[38]	54.55	25.38	1.41	10.14	6.44	0.60	0.70	0.10	-
[45]	55.94	24.83	0.82	10.02	5.89	0.30	0.22	-	-
[24]	51.7	25.2	0.6	0.05	2.5	0.3	3.7	-	0.2
[19]	54.6	21.1	1.5	5.6	7.5	1.3	0.4	0.3	-
[37]	54.86	22.39	1.27	6.05	13.72	0.32	0.60		9.60
[63]	53.92	21.13	1.55	11.19	11.11	0.40	0.24	0.14	0.32

## Data Availability

Not applicable.

## Abbreviations

AAM	Alkali-activated materials	PSD	Particle size distribution
AAS	Alkali-activated slag	SF	Silica fume
C	Cement	SSA	Specific surface area
DTG	Derivative thermogravimetric analysis	SS	Steel slag
EDS	Energy-dispersive spectroscopy	SCM	Supplementary cementitious material
FA	Fly ash	SEM	Scanning electron microscopy
FTIR	Fourier transform infrared spectroscopy	SAC	Sulfoaluminate cement
GGBS	Ground granulated blast slag	TIPA	Triisopropanolamine
LS	Lithium slag	TG	Thermogravimetry
LP	Limestone powder	TEA	Triethanolamine
MK	Metakaolin	UHPC	Ultra-high-performance concrete
NMR	Nuclear magnetic resonance	XRD	X-ray diffraction
OPC	Ordinary Portland cement	XRF	X-ray fluorescence
PCE	Polycarboxylate	XPS	X-ray photoelectron spectroscopy
